# Predictability of real temporal networks

**DOI:** 10.1093/nsr/nwaa015

**Published:** 2020-02-10

**Authors:** Disheng Tang, Wenbo Du, Louis Shekhtman, Yijie Wang, Shlomo Havlin, Xianbin Cao, Gang Yan

**Affiliations:** 1 School of Electronic and Information Engineering, Beihang University, Beijing 100191, China; 2 School of Physics Science and Engineering, Tongji University, Shanghai 200092, China; 3 National Engineering Laboratory of Big Data Application Technologies of Comprehensive Transportation, Beijing 100191, China; 4 Network Science Institute, Northeastern University, Boston, MA 02115, USA; 5 Department of Physics, Bar Ilan University, Ramat Gan 5290002, Israel; 6 Shanghai Institute of Intelligence Science and Technology, Tongji University, Shanghai 200092, China; 7 CAS Center for Excellence in Brain Science and Intelligence Technology, Chinese Academy of Sciences, Shanghai 200031, China

**Keywords:** temporal network, predictability, network entropy, predictive algorithm

## Abstract

Links in most real networks often change over time. Such temporality of links encodes the ordering and causality of interactions between nodes and has a profound effect on network dynamics and function. Empirical evidence has shown that the temporal nature of links in many real-world networks is not random. Nonetheless, it is challenging to predict temporal link patterns while considering the entanglement between topological and temporal link patterns. Here, we propose an entropy-rate-based framework, based on combined topological–temporal regularities, for quantifying the predictability of any temporal network. We apply our framework on various model networks, demonstrating that it indeed captures the intrinsic topological–temporal regularities whereas previous methods considered only temporal aspects. We also apply our framework on 18 real networks of different types and determine their predictability. Interestingly, we find that, for most real temporal networks, despite the greater complexity of predictability brought by the increase in dimension, the combined topological–temporal predictability is higher than the temporal predictability. Our results demonstrate the necessity for incorporating both temporal and topological aspects of networks in order to improve predictions of dynamical processes.

## Teaser

An entropy-based framework solved the long standing problem of quantifying the regularities in any temporal network whose links change over time, and unraveled the limit of predictability for 18 real temporal networks in diverse fields.

## INTRODUCTION

Link temporality describes the time-varying nature of couplings and interactions between nodes in real networks [1–12], which has been found to significantly affect network dynamics. Examples include innovative or epidemic diffusion [13], information aggregation [14], the emergence of cooperation [15] and the achievability of control [16]. Hence, in order to alter network dynamical states in a desirable way, it is essential to quantitatively understand both topological and temporal patterns. This raises a fundamental question: how predictable are real temporal networks? This question is much broader and distinct from time-series forecasting [17–19], which aims to predict the future evolution of *single* variables, and link prediction [20–23], the goal of which is to uncover the missing or future links in *static* networks. Here we offer an entropy-rate-based framework that considers the combined topology–temporal patterns and apply them to a wide range of model and real weighted and unweighted temporal networks, uncovering the prediction limits of real temporal networks.

## ANALYTICAL FRAMEWORK FOR PREDICTABILITY

**Figure 1.**
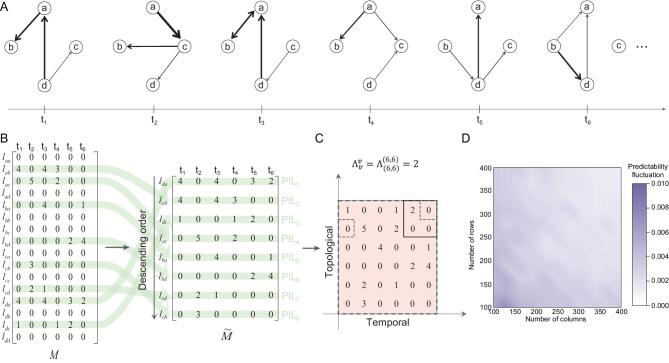
Quantifying the predictability of a temporal network. (A) The time-unfolded representation of a temporal network with four nodes. Each snapshot is a weighted directed network where the thickness of links represents their weights. (B) Matrix $M$ encodes the time evolution of each potential link, where each column embodies the structure of a snapshot. Links that rarely appear within the whole duration are removed from the matrix, resulting in matrix $\widetilde{M}$, which captures the meaningful part of $M$ (see the ‘Methods’ section). The rows of $\widetilde{M}$ are sorted into descending order according to the number of occurrences (see the ‘Methods’ section). A measure for the predictability of individual links (PIL) that captures only temporal correlations has been developed [28]. (C) Calculation of ${\rm{\Lambda }}_v^v$ for a part of $\widetilde{M}$. Note that ${\widetilde{M}_{C( k )}}$ is defined as a 2D square with side $k$, where $C ( k ) = \{ {v = ( {t,\ s} ) \in {{\boldsymbol{Z}}^2}:0 \le t \le k,0 \le s \le k} \}$ denotes the coordination set of elements in $\widetilde{M}$, then $\Lambda _v^v \equiv {{\rm inf}} \mathit\{ { {k \ge 1} |{{\widetilde{M}}_{u - C( k )}} \ne {{\widetilde{M}}_{v - C( k )}},\ \forall u \in [ {{\boldsymbol{0}},\ v} ],\ u \ne v} \}$. (D) The fluctuations of topological–temporal predictability (${\rm{TTP}}$) for different orders of rows in matrix $\widetilde{M}$. All matrices are extracted from a synthetic temporal network in Fig. 2A with rewiring probability$\ \ \ p\ = \ 0.5$. We also change $p$ and observe that it has no effect on the results (see Supplementary Material, Section V).

A temporal network with $n$ nodes consists of a series of snapshots (Fig. 1A), which can be described as a 2D expanded matrix $M$ (Fig. 1B). Each column in $M$ represents one snapshot and each row represents the temporality of a possible link, i.e. whether this link is present in a specific snapshot and its weight. Since all pairs of nodes must be taken into account, the number of rows in $M$ is ${n^2}$. The full information of the temporal network is encoded by this matrix $M$, which can be viewed as a stochastic vector process—a sequence of random vectors. To quantify the predictability of this vector process, we use the entropy rate, $H$, i.e. the asymptotic lower bound on the per-symbol description length [24], which is a rigorous measure of the level of randomness in the process. As illustrated in Fig. 1C, $H$ can be calculated using a generalized Lempel–Ziv algorithm [25], of which the essence is to calculate the recurrence times of different patterns within a square: a 2D square with side $k$ is defined as ${M_{C( k )}}$, where $C( k ) = \{ {v = ( {t,{\rm{\ }}s} ) \in {{\boldsymbol{Z}}^2}:0 \le t \le k,0 \le s \le k} \}$ and $v$ denotes the coordination of an element in $M$; ${\rm{\Lambda }}_v^v$ represents the smallest integer $k$ such that block ${M_{v - C( k )}}$ does not occur within the rectangle $( {{\boldsymbol{0}},\ v} ]$ except at position $v$, where ${\boldsymbol{0}} = ( {0,\ 0} )$. It has been proven [25] that $\mathop {\lim \inf }\limits_{n \to \infty } \frac{{{n^2}\! \log\! {n^2}}}{{\mathop \sum \nolimits_{v \in C( n )} {{( {{\rm{\Lambda }}_v^v} )}^2}}} \to H$. Thus, the entropy rate $H$ of the matrix $M$ is captured by

(1) \begin{equation*} H\! \left(M \right)\ = \frac{{{n^2}\! \log\!{n^2}}}{{\mathop \sum \nolimits_{v \in C\left( n \right)}\! {{\left( {{\rm{\Lambda }}_v^v} \right)}^2}}}, \end{equation*}

when the temporal network has a large number of snapshots (see Supplementary Material, Section II).

The predictability of a temporal network is the probability ${{\rm{\Pi }}_M}$ that a predictive algorithm can correctly forecast the future evolution of this network based on its history. Once we have the entropy rate $H( M )$, the upper bound of predictability ${\rm{\Pi }}_M^{{\rm{max}}}$ can be obtained by solving

(2) \begin{eqnarray*} H (M) &=& - \big({\rm{\Pi }}_M^{{\rm{max}}}{\rm{log}}({\rm{\Pi }}_M^{{\rm{max}}}) \nonumber\\ && +\, \left( {1 - {\rm{\Pi }}_M^{{\rm{max}}}} \right)\! {\rm{log}}\! \left( {1 - {\rm{\Pi }}_M^{{\rm{max}}}} \right) \big)\nonumber\\ && +\, \left( {1 - {\rm{\Pi }}_M^{{\rm{max}}}} \right){\rm{log}}\left( {N - 1} \right)\!, \end{eqnarray*}

where $N$ is the number of unique values in matrix $M$ (see the ‘Methods’ section and Supplementary Material, Section III). Here, ${\rm{\Pi }}_M^{{\rm{max}}}$ is the fundamental limit of predictability, i.e. in principle, no algorithm can predict the temporal network with an accuracy higher than ${\rm{\Pi }}_M^{{\rm{max}}}$. It is worth stressing that the entropy rate obtained with the generalized Lempel–Ziv algorithm is an asymptotic measure of randomness and Eq. (1) becomes more accurate when the number of time steps is larger. Hence, given the finite number of snapshots in real temporal network data sets, the calculated value of ${\rm{\Pi }}_M^{{\rm{max}}}$ should be interpreted as an asymptotic estimate of the upper bound of predictability. Moreover, ${\rm{\Pi }}_M^{{\rm{max}}}$ is an intrinsic property of the temporal network and does not depend on a specific predictive algorithm.

The snapshots of real temporal networks are usually very sparse, so most rows in $M$ consist of many zeros. Thus, we sort the rows, i.e. all potential links, in descending order according to the number of their occurrences in all snapshots and remove those links that are present in <10% of the snapshots, obtaining a new matrix $\widetilde{M}$ (see the ‘Methods’ section). Our analyses in both model and real networks show that the filtering process and the ordering of rows in $\widetilde{M}$ have a negligible effect on the predictability (see Fig. 1D, and also Supplementary Material, Sections IV and V); therefore, we use ${\rm{\Pi }}_{\widetilde{M}}^{{\rm{max}}}$ to quantify the predictability of temporal networks hereafter. Note that the original entropy rate (Eq. 1) applies to square matrices only, although the matrix $\widetilde{M}$ of a temporal network can be non-square. To overcome this issue, we split the original matrix into smaller squares with shorter history and find a linear relationship between the predictability and the number of squares, implying that longer history leads to higher predictability, allowing us to calculate the predictability of any temporal network (see the ‘Methods’ section and Supplementary Figs 1 and 2 in Supplementary Material, Section III).

## VALIDATION ON MODEL NETWORKS

**Figure 2.**
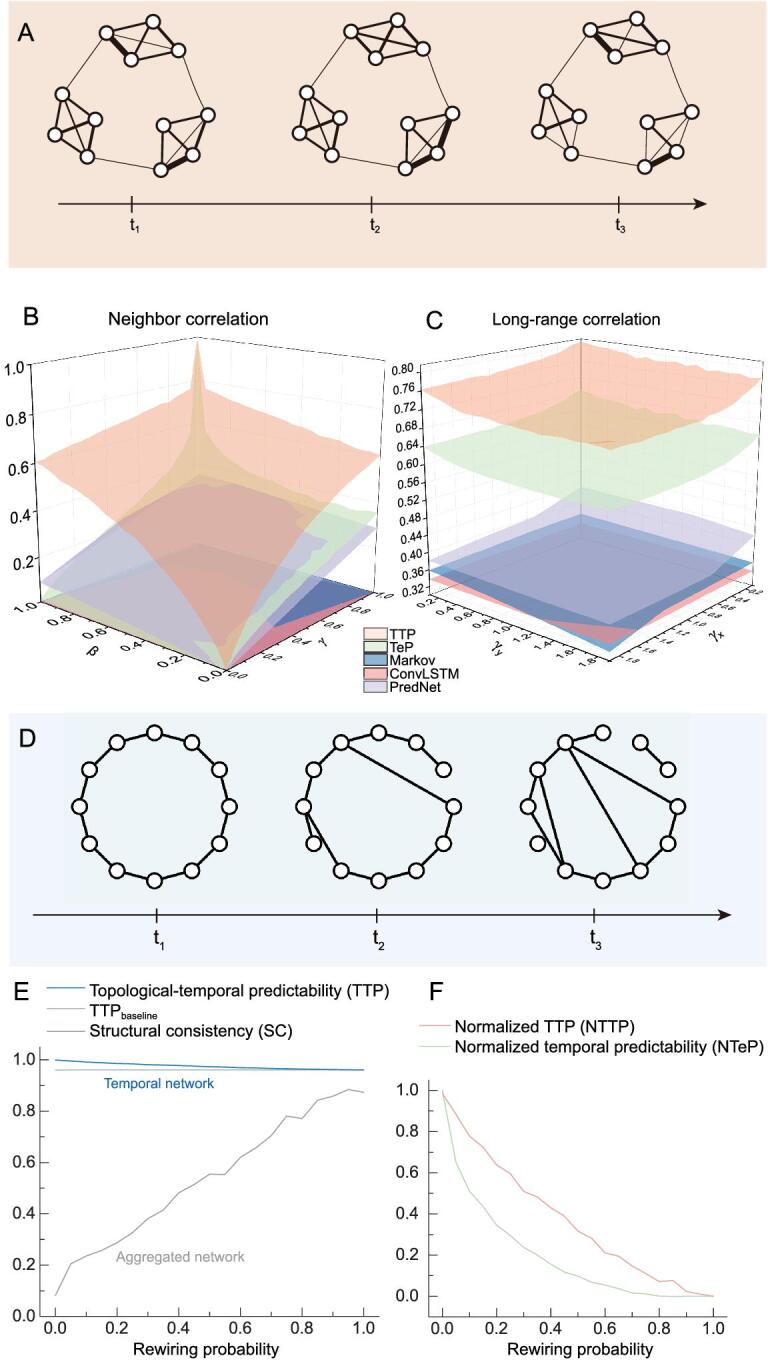
Predictability of synthetic temporal networks. (A) To test the impact of link weights on predictability, we develop a temporal stochastic block model with nearest-neighbor correlations (see Supplementary Material, Section VII for details) and long-range correlations [27]. The initial snapshot is generated by the stochastic block model [26], consisting of nodes uniformly assigned to specific communities. There are four communities with 100 nodes and 300 snapshots in each network, while the degree of each node is 3. The link weights in subsequent snapshots are generated according to topological parameters and temporal parameters for the two models, respectively, without changing the network topology. (B) Two predictability measures (TTP and TeP) with three predictive algorithms (Markov [29], ConvLSTM [30] and PredNet [31]) on nearest-neighbor correlations with topological parameter $\beta $ and temporal parameter $\gamma $. Maximum of $\beta $ or $\gamma $ means the strongest memory in the topological or temporal dimension. ${\rm{TeP}}$ is obtained by averaging PIL. (C) Two predictability measures with three predictive algorithms on long-range correlations with a power-law correlation function $C ( {r,\ \varphi } ) = {r^{\!- {\gamma _x}}} {\rm cos}^{2}\varphi + {r^{\! - {\gamma _y}}} {\rm sin}^{2}\varphi $, where $( {r,\ \varphi } )$ are polar coordinates and ${\gamma _x}$ is regarded as the temporal parameter, while ${\gamma _y}$ is the topological dimension. Results are averaged over 10 independent realizations of the networks. (D) To test the impact of network topology on predictability, we develop an evolving small-world network model. The first snapshot is a ring network; subsequent topologies of the network are generated by randomly rewiring a fraction $p$ of links in the previous snapshot. (E, F) Predictabilities of evolving small-world networks against rewiring probability. The networks are generated by the model in (D) with 50 nodes and average degree 2. Structural consistency (SC) is an existing predictability measure for static undirected and unweighted networks [32]. We normalize ${\rm{TTP}}$ over the ${\rm{TT}}{{\rm{P}}_{{\rm{bl}}}}$ to eliminate the impact of link sparsity (see the ‘Methods’ section), obtaining the intrinsic predictability of a temporal network, and also obtain normalized TeP for comparison (see the ‘Methods’ section).

Next, we test and validate our measure, ${\rm{\Pi }}_{\widetilde{M}}^{{\rm{max}}}$, i.e. the topological–temporal predictability (TTP), in synthetic weighted temporal networks (Fig. 2A). The initial snapshot is a network with communities generated by a stochastic block model [26] with links assigned with random weights. In each snapshot henceforth, to generate a neighbor correlation for each link, we activate either the temporal parameter $\gamma $ or the structural parameter $\beta $. With probability $\beta $, we modify the structure and the link changes its weight to that of an adjacent link; with probability $\gamma $, we modify the temporal aspect and the link weight stays the same as in the last snapshot; otherwise, the link is assigned a random value (see the ‘Methods’ section). Long-range correlations are generated through 2D fractional Gaussian noise (FGN) [27], with a power-law correlation function $C ( {r,\ \varphi } ) = {r^{\! - {\gamma _x}}} {\rm cos}^{2}\varphi + {r^{\! - {\gamma _y}}} {\rm sin}^{2}\varphi $, where $( {r,\ \varphi } )$ are polar coordinates and ${\gamma _x}$ is regarded as a decay parameter in the temporal dimension, while ${\gamma _y}$ is for the topological dimension. We compare, in Fig. 2B and C, our TTP with an existing measure, namely temporal predictability (TeP) [28], which considers the links of a temporal network as merely a set of uncorrelated time series and captures only the temporal regularity (see the ‘Methods’ section), and also with three predictive algorithms. For this, we employ three commonly used methods, namely Markov [29], ConvLSTM [30] and PredNet [31], to forecast the future evolution of real networks. Markov considers a temporal network as a set of uncorrelated time series, ConvLSTM takes into consideration link correlations, and PredNet is a dynamic matrix-prediction algorithm based on ConvLSTM (see Supplementary Material, Section IX for details). As shown in Fig. 2B, TeP is significantly smaller than TTP and, when parameters $\beta $ and $\gamma $ increase, TeP can be seen to be nearly independent of the structural parameter $\beta $, due to the fact that TeP only partially characterizes the regularity of temporal networks. Note that, since $\beta $ and $\gamma $ are not completely independent of each other, TTP is still slightly higher than TeP even when $\beta $ and $\gamma $ approach zero (highest correlations). The higher accuracy of PredNet than the upper bound of predictability provided by TeP, as well as the poor performance of Markov, both indicate the significance of topological information. The unexpected insufficient performance of ConvLSTM, however, is caused by the deconvolution layer, which introduces errors. We further find similar results for 2D FGN, although the varying range of predictabilities is much smaller due to fewer possible values.

We also introduce synthetic unweighted temporal networks (Fig. 2D) to validate our measure (TTP). The initial snapshot is a ring and each snapshot thereafter is generated by randomly rewiring a fraction $p$ of links in the most recent snapshot. Obviously, as $p$ increases, the network becomes more random, and hence less predictable (see Supplementary Material, Section VI). However, the structural consistency of the aggregated network—a measure that captures only topological regularity on static networks [32]—leads to conflicting increasing predictability (Fig. 2E), demonstrating again the necessity for considering link temporality.

In contrast, our measure, TTP, decreases monotonously when $p$ increases. Yet, due to the high sparsity of these temporal networks, TTP remains high for all values of $p$. To remove the impact of sparsity, we define and calculate the normalized topological–temporal predictability $({{\rm{NTTP}}}) = ({{\rm{TTP}}} - {{\rm{TT}}{{\rm{P}}_{{\rm{bl}}}}})/( {1.0} - {\rm{TT}{{\rm{P}}_{{\rm{bl}}}}})$ for ${\rm{TT}}{{\rm{P}}_{{\rm{bl}}}}$ < 1.0 (see the ‘Methods’ section), where NTTP is the normalized TTP and ${\rm{TT}}{{\rm{P}}_{{\rm{bl}}}}$ is the TTP of the shuffled network, which can be viewed as the lower bound of the predictability of temporal networks. In comparison with NTTP, we also normalize TeP (called here the normalized temporal predictability (NTeP)) over shuffled links (see the ‘Methods’ section). As shown in Fig. 2F, for $p\, =\, 0$, the network is fully predictable (${\rm{NTTP}} \approx 1.0$) and, for $p\, =\, 1.0,$ the network becomes totally random and unpredictable (NTTP vanishes). Even though NTeP has the analogous decreasing behavior, it is usually lower than NTTP due to the lack of topological information. Therefore, the NTTP indeed captures the intrinsic regularity of temporal networks.

## PREDICTABILITY AND PREDICTIVE ALGORITHMS ON REAL NETWORKS

**Figure 3.**
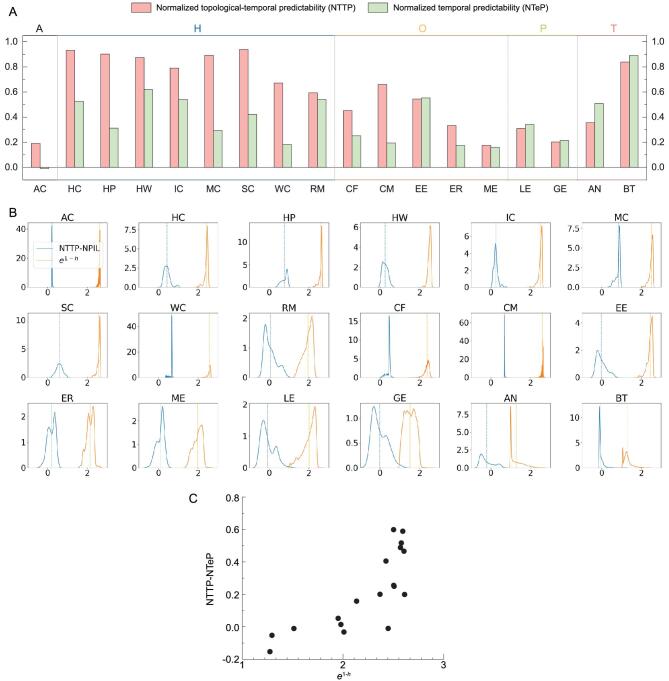
Predictability of real temporal networks. (A) ${\rm{NTTP}}$ and ${\rm{NTeP}}$ for 18 real networks (see Supplementary Material, Section I for the description of these network datasets). ‘A’ means animal contacts, ‘H’ denotes human contacts, ‘O’ means online communications, ‘P’ represents political events, and ‘T’ stands for transportation. Note that NTeP can be higher than NTTP because the baselines for these two normalizations are different. (B) Distributions of NTTP-NPIL and ${e^{1 - h}}$ on real-world networks, where NPIL is the normalized predictability of individual links (see the ‘Methods’ section) and $h$ is the normalized Hamming distance between each link pair. (C) Correlation of average of NTTP-NPIL and ${e^{1 - h}}$ for 18 real networks.

We apply our framework on 18 real temporal networks in diverse scenarios, including animal interactions, human contacts, online communications, political events and transportation (see Supplementary Material, Section I for the description of these network datasets). We group these networks into five categories and reveal the intrinsic predictability profile, consisting of NTTP and NTeP, for each network (Fig. 3A). We find that human contacts have the highest averaged NTTP, probably resulting from their synchronized bursty nature, while temporal regularities dominate the overall predictability of transportation networks due to the periodicity of each link (see Supplementary Material, Section VIII for details). Since the baselines for the normalizations in NTTP and NTeP are different, NTeP can be higher than NTTP. However, we find another interesting phenomenon in most networks (excluding Enron-Email (EE), Levant-Event (LE), Aviation-Network (AN) and Britain-Transportation (BT)): the intrinsic combined predictability is higher than TeP despite the greater complexity of capturing 2D regularity rather than 1D. This implies the significance of the topological information as well as the correlation between the temporal and topological patterns. Surprisingly, we also find strong correlations between topological regularity (characterized by the Hamming distance between each link pair) and the difference between TTP and NPIL (normalized predictability of individual links) (see Fig. 3B and C), suggesting that the intrinsic predictability of real networks mostly originates from temporal and topological regularity, rather than from the interdependence between them.

**Figure 4.**
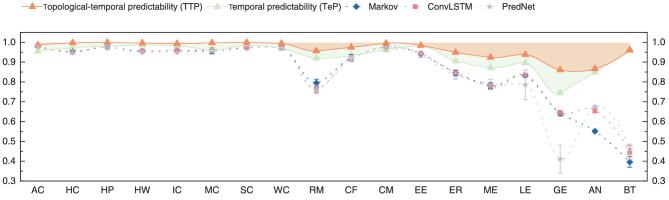
Predictive power of existing algorithms. Markov considers a temporal network as a set of uncorrelated time series [29], ConvLSTM takes into consideration of link correlations [30], and PredNet is a dynamic matrix-prediction algorithm based on ConvLSTM [31] (see Supplementary Material, Section IX for details). Error bars are the standard deviation of each algorithm over 10 different runs. Note that all algorithms do not reach our topological–temporal predictabilities of real temporal networks. The accuracy of at least one algorithm is higher than TeP on AC, Marseilles-Contact (MC), Workplace-Contact (WC), College-Forum (CF) and CM networks.

Next, we compare our measure to the predictive power of existing algorithms. We find that the above existing algorithms mostly fall short in prediction (see Fig. 4). Indeed, for a few networks (Ant-Colony (AC): $p\hbox{-}{\rm{value\ }} = \ 7.1\ \times {10^{ - 15}}$, College-Message (CM): $p\hbox{-}{\rm{value\ }} = \ 6.1\ \times {10^{ - 7}}$), their accuracy is higher than the maximum predictability found by ${\rm{TeP}}$. This is probably because TeP fails to incorporate the topological aspects. However, we found that the predictability given by our TTP measure always remains out of reach from the current algorithms, indicating again that TeP alone cannot characterize the regularities in temporal networks.

## DISCUSSION AND OUTLOOK

We developed a 2D framework, based on combined topology–temporal features, for quantifying the intrinsic predictability and uncovering the predictability profile of any temporal network. Importantly, we find that the accuracy of current algorithms could be higher than the current temporal-only predictability methods for some real temporal networks. Furthermore, they never exceed our TTP measure. Given the fact that predictability is an essential property of temporal networks, our findings suggest that more accurate predictive algorithms are needed to capture the regularities of real temporal networks, i.e. there is room for researchers to continue improving their predictive algorithms. In addition, applying our measure of predictability to detect the changing points of temporal networks and systematically investigating the impact of predictability on dynamical processes and control on temporal networks are worth future pursuits.

## METHODS

### Matrix filtering

As mentioned above, most rows in matrix $M$ for a real temporal network consistently remain zero. Such link sparsity leads to high predictability. We sort links ${\ell _1},{\ell _2}, \ldots ,{\ell _n}$ in the matrix $M$ by their activation rates ${a_1},{a_2}, \ldots ,{a_n}$ in descending order, hence ${a_1} > {a_2} > \ldots {a_n}$, then obtain $\widetilde{M}$ according to the filtering rules:

$$\begin{equation*}
\widetilde{M} = \left\{\begin{array}{@{}*{1}{c}@{}} {\left\{ {{\ell _1},{\ell _2}, \ldots ,{\ell _m}} \right\}|m = \inf\! \left\{ {m \in \left( {1,2, \ldots ,n} \right)|\sum\limits_{i=1}^m {{a_i} \ge 0.6} \sum\limits_{i=1}^n {{a_i}} } \right\},\ m < {m_\theta }}\\ {\left\{ {{\ell _1},{\ell _2}, \ldots ,{\ell _m}} \right\}|m = \inf \left\{ {m \in \left( {1,2, \ldots ,n} \right)|{a_m} \ge 0.1} \right\},\quad\quad\quad\quad m \ge {m_\theta}}
\end{array}\,. \right.\
\end{equation*}$$

Since the estimation of the entropy rate converges to the real entropy when the size of the matrix goes to infinity [25], we include at least ${m_\theta }$ most active links or 60% of non-zero elements in the matrix to diminish errors. Due to computational restrictions, we set ${m_\theta } = 1000$, although, in principle, it could be higher with sufficient resources. All the calculations are performed on $\widetilde{M}$. We show that the matrix-filtering method has no influence on the results after reducing the matrix $M$ to $\widetilde{M}$ in Supplementary Material, Section IV.

### Derivation of predictability and its normalization

Although a detailed derivation is provided in Supplementary Material, Section II, here we adumbrate the main steps used to derive the upper bound of predictability. The entropy rate of a temporal network, which is characterized as a random field, is defined as

$$\begin{equation*}\begin{array}{@{}*{2}{l}@{}} {H\! ( M )}& {\equiv \mathop {\lim }\limits_{\begin{array}{@{}*{1}{c}@{}} \scriptstyle{L\! \to\! \infty }\\ \scriptstyle{T\! \to\! \infty} \end{array}} \displaystyle\frac{1}{{LT}}H\! \left( {{M^{LT}}} \right)}\\ {}&\,\,{= \mathop {\lim }\limits_{\begin{array}{@{}*{1}{c}@{}} \scriptstyle{L\! \to\! \infty }\\ \scriptstyle{T \to \infty } \end{array}} \displaystyle\frac{1}{{LT}}\mathop \sum \limits_{\begin{array}{@{}*{1}{c}@{}} \scriptstyle{1\, \le\, l\, \le\, L}\\ \scriptstyle{1\, \le\, t\, \le\, T} \end{array}} \,\,H\! \left({{M_{lt}}\left| {\mathit {\rm history}\ \mathit {\rm of}\ {M_{lt}}} \right.}\! \right)}\\ {}&\,\,{{\rm{\ }} = \mathop {\lim }\limits_{\begin{array}{@{}*{1}{c}@{}} \scriptstyle{L\! \to\! \infty }\\ \scriptstyle{T\! \to\! \infty} \end{array}} \displaystyle\frac{1}{{LT}}\mathop \sum \limits_{\begin{array}{@{}*{1}{c}@{}} \scriptstyle{1\, \le\, l\, \le\, L}\\ \scriptstyle{1\, \le\, t \le\, T} \end{array}} H\! \left({l,t} \right)}\, ,
\end{array}
\end{equation*}$$

where $\mathit{\rm history}\ \mathit {\rm of}\ {M_{lt}} \equiv \{{{M_{ij}}:\ ({j < t})\ \mathit {\rm or}}$${\ ({j = t\ \mathit {\rm and}\ i < l})}\}$. Suppose $P\! ({{M_{lt}} = {{\widehat{M}}_{lt}}\ | {{\tau _{lt}}}})$ is the probability that is based on the history ${\tau _{lt}}$, the actual value of ${M_{lt}}$ agrees with our estimation$\ {\widehat{M}_{lt}}$, and $\lambda ( {{\tau _{lt}}})$ is the probability that $\ {M_{lt}}$ takes the most likely value given ${\tau _{lt}}$, thus

$$\begin{equation*}
\lambda\! \left({{\tau _{lt}}} \right) \equiv {\rm max} \left\{{P\! \left({{M_{lt}} = {{\widehat{M}}_{lt}}\ \left| {{\tau _{lt}}} \right.} \right)} \right\}\!.
\end{equation*}$$

Let $P\! ({{\tau _{lt}}})$ be the probability of observing a specific history. It can be demonstrated that the best prediction strategy based on this history is to adopt the most likely value [28]; thus, the predictability of ${M_{lt}}$ is

$$\begin{equation*}
{{\rm{\Pi}}_M}\! \left({l,t} \right) \equiv \mathop \sum \limits_{{\tau _{lt}}} P\! \left({{\tau _{lt}}} \right)\!\lambda\! \left({{\tau _{lt}}} \right)\!.\end{equation*}$$

Then the overall predictability ${\rm{\Pi}}$ of a random field is

$$\begin{equation*}
{{\rm{\Pi }}_M}\! \equiv \mathop {{\rm{lim}}}\limits_{\begin{array}{@{}*{1}{c}@{}} \scriptstyle{L\! \to\! \infty }\\ \scriptstyle{T\! \to\! \infty } \end{array}} \displaystyle\frac{1}{{LT}}\mathop \sum \limits_{\begin{array}{@{}*{1}{c}@{}} \scriptstyle{1\, \le\, l\, \le\, L}\\ \scriptstyle{1\, \le\, t\, \le\, T} \end{array}} {{\rm{\Pi }}_M}\! \left({l,t} \right).\end{equation*}$$

Because the entropy increases as the distribution becomes uniform, the distribution created by setting the remaining probabilities to be the same while preserving the most likely value $\lambda ({{\tau _{lt}}}) = {p_{\rm max}}$ has an entropy no less than the original distribution. Note that ${M_v} \in \mathcal{A}$, ${M_v} \equiv |\mathcal{A}|$ and denote $N \equiv | \mathcal{A} |$. The entropy of the new distribution is

$$\begin{eqnarray*}
H\! (M) = \nonumber\\
-\, ( {{\rm{\Pi }}_M^{{\rm{max}}}{\rm log}{\rm{\Pi }}_M^{\rm max} + ({1 - {\rm{\Pi }}_M^{\rm max}}) {\rm log} ({1 - {\rm{\Pi }}_M^{\rm max}})}) \nonumber\\
+\, ({1 - {\rm{\Pi}}_M^{\rm max}}) {\rm log} ({N - 1}).
\end{eqnarray*}$$

Then the solution of ${\rm{\Pi }}_M^{\rm max}$ in the above equation is the upper bound of predictability ${{\rm{\Pi }}_M}$. We adopt the entropy estimator [25] as the entropy rate$\ H( M )$ for the calculation of predictability's upper bound ${\rm{\Pi }}_M^{\rm max}$

$$\begin{equation*}
H\! \left(M \right) = \frac{{{n^2}\! \log {n^2}}}{{\mathop \sum \nolimits_{v \in C\left( n \right)}\! {{\left( {{\rm{\Lambda }}_v^v} \right)}^2}}}\ ,\end{equation*}$$

where $v\ = ( {{v_1},\ {v_2}})$, a 2D square with side $k$, is defined as ${M_{C( k )}}$, $C ( k ) = \{{v = ( {{v_1},\ {v_2}} ) \in {{\boldsymbol{Z}}^2}:0 \le {v_i} \le k,\ {\rm{for\ all}}\ i}\}$ and ${\rm{\Lambda }}_v^v$ denotes the smallest integer$\ k$ such that block ${M_{v - C( k )}}$ does not occur within the rectangle $( {{\boldsymbol 0},\ v}]$ except at position $v$.

The link sparsity of a temporal network largely determines its predictability even after we adopt matrix filtering. To remove the impact of sparsity and obtain the intrinsic predictability of a temporal network, we normalize ${\rm{\Pi }}_M^{\rm max}$ over the baseline (i.e. predictability of shuffled network), which captures only the regularity in the link-weight distribution. Therefore, we have

$$\begin{equation*}{p_{{\rm{norm}}}} = \left\{ {\begin{array}{@{}*{1}{c}@{}} {1,\ b = 1\ {\rm and}\ p = 1}\\ {\displaystyle\frac{{p - b}}{{\left( {1 - b} \right)}},\ {\rm otherwise}} \end{array}} \right.,\end{equation*}$$

where $p$ is the original predictability and $b$ is the baseline. It is worth noting that the NTeP is the average of the NPIL, which is obtained by normalizing PIL over its own baseline ${\rm{PI}}{{\rm{L}}_{{\rm{bl}}}}$.

### Generalization of predictability using predictive congruency

Since the application of our entropy estimator is limited to only square matrices [25], we explore the correlation of weighted-average predictability and number of squares by gradually splitting the non-square matrix into a set of units, i.e. $1\ \times \ 1$ squares, and compute the predictability of each square. We find the linear relationship between the weighted-average predictability and the number of squares, including units. Assume matrix $\widetilde{M}$ is split into $Q$ squares ${s_1},\ {s_2},\ \ldots ,\ {s_Q}$ in the first splitting stage, along with $u$ units; let ${e_{{s_1}}},\ {e_{{s_2}}}\!,\ \ldots ,\ {e_{{s_Q}}}$ be the sizes of squares, then the areas of the squares are ${e_{{s_1}}}^2,\ {e_{{s_2}}}^2,\ \ldots ,\ {e_{{s_Q}}}^2$. It is worth noting that we define the predictability of units as $\frac{1}{{| \mathcal{A} |}}$, where $\mathcal{A}$ is the finite value set of link weights in the temporal network. Then the weighted-average predictability at stage $i$, of which the weight equals the portion of corresponding square in the matrix, is defined as

$$\begin{equation*}
{p_i} = \left( {\mathop \sum \limits_{j=1}^{Q - i + 1} {e_{\!{s_{\! j\!}}}}^2\!{p_{{s_{\!j}}}} + \frac{{\mathop \sum \nolimits_{Q - i + 2}^Q\! {e_{{\! s_{\! j}}}}^2 + u}}{{\left| \mathcal{A} \right|}}} \right) /D,
\end{equation*}$$

while the number of squares for splitting stage $i$ is

$$\begin{equation*}{N_i} = Q - i + 1 + \mathop \sum \limits_{j=Q - i + 2}^Q {e_{{\! s_{\!j}}}}^2 + u\!.\end{equation*}$$

Note that ${N_1} = Q + u$. Since there is a linear relationship between ${p_i}$ and ${N_i}$, thus

$$\begin{equation*}\frac{{{e_{{\! s_{\! i\!}}}}^2}}{{{e_{{\! s_{\!i\!}}}}^2 - 1}}\ \left( {\frac{1}{{\left| \mathcal{A} \right|}} - {p_i}} \right) = \ kD,\end{equation*}$$

$$\begin{equation*}{p_i} = \ k{N_i} + b,\end{equation*}$$

where $1 \le i \le Q - 1$, $k$ and $b$ are constants. According to our observations of $k < 0$, the negative linear relationship between ${p_i}$ and ${N_i}$ indicates the positive correlation between the length of memory and predictability, since a smaller ${N_i}$ means larger squares with more memory. We define this as predictive congruency and use it to obtain the TTP of each temporal network with non-square matrix

$$\begin{equation*}p = {p_{{N_i} = 1}} = k + b.\end{equation*}$$

### Synthetic networks

The temporal stochastic block model is used to test the impact of link weight while topology remains invariant. When generating neighbor correlation in a temporal stochastic block model, we determine the weight of links individually according to parameters $\beta $ and $\gamma $. For each link, there is a probability of modifying the link weight based on structural or temporal aspects. If the structural parameter is selected, then the probability for the link to adopt the same weight as its neighboring link is $\beta $; when the temporal parameter is activated, the probability for the link to remain the same as the previous snapshot equals $\gamma $. Otherwise, the link is assigned a random value. Suppose there are $m$ links ${\ell _1},{\ell _2}, \ldots ,{\ell _n}$ in the matrix; then, the probability density function of a link weight at a certain time is

$$\begin{eqnarray*}
f\!\! \left({{\ell _i}\!\! \left(t \right)} \right) &=& {p_\beta } \beta {\delta _{{\ell _i}\! \left( t \right){\ell _j}\left( t \right)}} + {p_\gamma}\!\gamma\! {\delta _{{\ell _i}\! \left( t \right){\ell _i}\! \left( {t - 1} \right)}} \nonumber\\
+\, \left( {1 - {p_\beta }\beta - {p_\gamma }\gamma } \right){\delta _{{\ell _i}\! \left( t \right)r}},
\end{eqnarray*}$$

where $i, j \in [ {1, n} ], i \ne j$. ${p_\beta }$ and ${p_\gamma }$ are the probabilities to choose the structural parameter $\beta $ and temporal parameter $\gamma $, respectively, ${p_\beta } + \ {p_\gamma } = \ 1$. ${\delta _{xy}}$ is the Kronecker delta function, and $r$ is a random number.

Specifically, we assume ${p_\beta } = {p_\gamma }\ = \ 0.5$ and generate the matrix column by column, from top to bottom within each column. If a link is determined to adopt the weight of its neighboring link at a certain time, we assign the prior link weight to it, which is its adjacent element in the matrix. The initial snapshot of the evolving small-world model is a ring network; then, we obtain each snapshot by rewiring a fraction of links in the previous snapshot.

### TeP and NTeP

TeP is the average PIL in the network (Fig. 1). To eliminate the influence of sparsity, we also normalize PIL over its own baseline to obtain NPIL, and NTeP as the average of NPIL.

$$\begin{equation*}
{\rm{TeP}}\ = \langle {\rm{PIL}}\rangle,
\end{equation*}$$

$$\begin{equation*}
{\rm{NPIL}} = \left\{{\begin{array}{@{}*{2}{l}@{}} {1,}&{{\rm{PI}}{{\rm{L}}_{{\rm{bl}}}} = 1\,{\rm{and}}\,{\rm{PIL}} = 1}\\ {\displaystyle\frac{{{\rm{PIL}} - {\rm{PI}}{{\rm{L}}_{{\rm{bl}}}}}}{{\left( {1 - {\rm{PI}}{{\rm{L}}_{{\rm{bl}}}}} \right)}},}&{{\rm{otherwise}}}\,, \end{array}} \right.\end{equation*}$$

$$\begin{equation*}
{\rm{NTeP}} = \langle {\rm{NPIL}}\rangle ,\end{equation*}$$

where ${\rm{PI}}{{\rm{L}}_{{\rm{bl}}}}$ is the ${\rm{PIL}}$ of shuffled links.

## Supplementary Material

nwaa015_Supplemental_File

## References

[1] Holme P , Saramäki J. Temporal networks. Phys Rep 2012; 519: 97–125.

[2] Kossinets G , Watts DJ. Empirical analysis of an evolving social network. Science 2006; 311: 88–90. 16400149 10.1126/science.1116869

[3] Mucha PJ , Richardson T, Macon K et al. Community structure in time-dependent, multiscale, and multiplex networks. Science 2010; 328: 876–8. 20466926 10.1126/science.1184819

[4] Masuda N , Klemm K, Eguíluz VM. Temporal networks: slowing down diffusion by long lasting interactions. Phys Rev Lett 2013; 111: 188701. 24237569 10.1103/PhysRevLett.111.188701

[5] Majdandzic A , Podobnik B, Buldyrev SV et al. Spontaneous recovery in dynamical networks. Nat Phys 2014; 10: 34–8.

[6] Scholtes I , Wider N, Pfitzner R et al. Causality-driven slow-down and speed-up of diffusion in non-Markovian temporal networks. Nat Commun 2014; 5: 5024. 25248462 10.1038/ncomms6024

[7] Génois M , Vestergaard CL, Cattuto C et al. Compensating for population sampling in simulations of epidemic spread on temporal contact networks. Nat Commun 2015; 6: 8860. 26563418 10.1038/ncomms9860 PMC4660211

[8] Peixoto TP , Rosvall M. Modelling sequences and temporal networks with dynamic community structures. Nat Commun 2017; 8: 582. 28928409 10.1038/s41467-017-00148-9 PMC5605535

[9] Valencia M , Martinerie J, Dupont S et al. Dynamic small-world behavior in functional brain networks unveiled by an event-related networks approach. Phys Rev E 2008; 77: 050905. 10.1103/PhysRevE.77.050905 18643019

[10] Dimitriadis SI , Laskaris NA, Tsirka V et al. Tracking brain dynamics via time-dependent network analysis. J Neurosci Methods 2010; 193: 145–55. 20817039 10.1016/j.jneumeth.2010.08.027

[11] Hidalgo CA , Blumm N, Barabási A-L et al. A dynamic network approach for the study of human phenotypes. PLoS Comput Biol 2009; 5: e1000353. 19360091 10.1371/journal.pcbi.1000353 PMC2661364

[12] Li X , Li X. Reconstruction of stochastic temporal networks through diffusive arrival times. Nat Commun 2017; 8: 15729. 10.1038/ncomms15729 PMC5472785 28604687

[13] Vazquez A , Racz B, Lukacs A et al. Impact of non-Poissonian activity patterns on spreading processes. Phys Rev Lett 2007; 98: 158702. 17501392 10.1103/PhysRevLett.98.158702

[14] Jelasity M , Montresor A, Babaoglu O. Gossip-based aggregation in large dynamic networks. ACM T Comput Syst 2005; 23: 219–52.

[15] Rand DG , Arbesman S, Christakis NA. Dynamic social networks promote cooperation in experiments with humans. Proc Natl Acad Sci USA 2011; 108: 19193–8. 22084103 10.1073/pnas.1108243108 PMC3228461

[16] Li A , Cornelius SP, Liu YY et al. The fundamental advantages of temporal networks. Science 2017; 358: 1042–6. 29170233 10.1126/science.aai7488

[17] Brockwell PJ , Davis RA, Calder MV. Introduction to Time Series and Forecasting. New York: Springer, 2002.

[18] Chatfield C . Time-Series Forecasting. London: Chapman and Hall/CRC, 2000.

[19] Wang D , Song C, Barabási A-L. Quantifying long-term scientific impact. Science 2013; 342: 127–32. 24092745 10.1126/science.1237825

[20] Clauset A , Moore C, Newman MEJ. Hierarchical structure and the prediction of missing links in networks. Nature 2008; 453: 98–101. 18451861 10.1038/nature06830

[21] Kovács IA , Luck K, Spirohn K et al. Network-based prediction of protein interactions. Nat Commun 2019; 10: 1240. 30886144 10.1038/s41467-019-09177-y PMC6423278

[22] Cheng F , Kovács IA, Barabási A-L. Network-based prediction of drug combinations. Nat Commun 2019; 10: 1197. 30867426 10.1038/s41467-019-09186-x PMC6416394

[23] Liben‐Nowell D , Kleinberg J. The link‐prediction problem for social networks. J Am Soc Inf Sci Technol 2007; 58: 1019–31.

[24] Anastassiou D , Sakrison D. Some results regarding the entropy rate of random fields (Corresp.). IEEE Trans Inf Theory 1982; 28: 340–3.

[25] Kontoyiannis I , Algoet PH, Suhov YM et al. Nonparametric entropy estimation for stationary processes and random fields, with applications to English text. IEEE Trans Inf Theory 1998; 44: 1319–27.

[26] Holland PW , Laskey KB, Leinhardt S. Stochastic blockmodels: first steps. Soc Networks 1983; 5: 109–37.

[27] Makse HA , Havlin S, Schwartz M et al. Method for generating long-range correlations for large systems. Phys Rev E 1996; 53: 5445–9. 10.1103/physreve.53.5445 9964877

[28] Song C , Qu Z, Blumm N et al. Limits of predictability in human mobility. Science 2010; 327: 1018–21. 20167789 10.1126/science.1177170

[29] Kemeny JG , Snell JL. Markov Chains. New York: Springer-Verlag, 1976.

[30] Shi X , Chen Z, Wang H et al. Convolutional LSTM network: a machine learning approach for precipitation nowcasting. In: Cortes C, Lawrence ND, Lee DD et al. (eds.). Advances in Neural Information Processing Systems. Montreal: Neural Information Processing Systems Foundation, 2015, 802–10.

[31] Lotter W , Kreiman G, Cox D. Deep predictive coding networks for video prediction and unsupervised learning. arXiv:160508104.

[32] Lü L , Pan L, Zhou T et al. Toward link predictability of complex networks. Proc Natl Acad Sci USA 2015; 112: 2325–30. 25659742 10.1073/pnas.1424644112 PMC4345601

