# Supplementary material for: Predictability of real temporal networks
Source: Natl Sci Rev. 2020 Feb 10;7(5):929–37. doi: 10.1093/nsr/nwaa015 (PMC8288877; doi:10.1093/nsr/nwaa015)
Supplement: nwaa015_Supplemental_File [file nwaa015_supplemental_file.docx]

# Supplementary Materials for Predictability of real temporal networks

**CONTENTS**

[I. Datasets 2](#_Toc30713469)

[II. Predictability 4](#_Toc30713470)

[III. Generalization and predictive congruency 6](#_Toc30713471)

[IV. Matrix shuffling and filtering 10](#_Toc30713472)

[V. Impact of row orders 12](#_Toc30713473)

[VI. Impact of snapshot duration 16](#_Toc30713474)

[VII. NTTP of incomplete data 17](#_Toc30713475)

[VIII. NTTP of Submatrices 18](#_Toc30713476)

[IX. Characteristics of real temporal networks 20](#_Toc30713477)

[X. Graphic presentation of model networks 23](#_Toc30713478)

[XI. Predictive algorithms 24](#_Toc30713479)

[References 28](#_Toc30713480)

### I. Datasets

In the main text we applied our framework to 18 real temporal networks. Ant-Colony (AC) records the interactions between 218 ants (*1*); Aviation-Network (AN) represents the domestic schedule of 224 airports in China; Britain-Transportation (BT) contains the schedules of 5 different types of transportation in the United Kingdom, where the nodes represent the airports or stations and links stand for the traffic flow (*2*); College-Forum network (CF) records user posts and replies in the forum of a university (*3*); College-Message dataset (CM) consists of messages in an online social network at a university (*4*); Enron-Email (EE) collects the emails between 150 employees in Enron Corporation (*5*); European-Research network (ER) is generated using email data from a large European research institution (*6*); Manufacturing-Email network (ME) is an internal email communication network between employees of a mid-sized manufacturing company (*7*); Gulf-Event network (GE) represents political actions between 200 countries and areas; Levant-Event (LE) contains political actions between 462 countries and areas; Haggle-Contact (HC) records the contacts between people measured by carried wireless devices (*8*); Hypertext-Proximity (HP) is a human contact network where each node represents a person and the links between them represent proximity (*9*); Hospital-Ward network (HW) is the temporal network of contacts between patients, patients and workers and among workers in a hospital ward (*10*); Infectious-Contact (IC) records face-to-face behaviors of people during the exhibition INFECTIOUS (*9*); Marseilles-Contact (MC) contains the temporal contacts between students in a high school in Marseilles in 2012 (*11*); Reality-Mining data (RM) is the recording of human contacts among 87 students in MIT (*12*); Student-Contact (SC) is the temporal network of contacts between students in a high school in Marseilles in 2011 (*11*); Workplace-Contact dataset (WC) is the temporal network of contacts between individuals in an office building in France (*13*). The link weight in datasets GE and LE means specific political strategy, while it represents the interaction frequency in other networks.

| **Datasets** | **Category** | **Type** | **Nodes** | **Links** | **Shape of** $\tilde{\boldsymbol{M}}$ | **Link Density** | **Duration** |
| --- | --- | --- | --- | --- | --- | --- | --- |
| Ant Colony (AC) | A | directed | 278 | 4542 | (1255, 176) | 0.021 | 10S |
| Haggle Contact (HC) | H | undirected | 274 | 11956 | (446, 413) | 0.065 | 10m |
| Hypertext Proximity (HP) | H | undirected | 112 | 3746 | (434, 347) | 0.025 | 10m |
| Hospital Ward (HW) | H | undirected | 75 | 4642 | (183, 573) | 0.044 | 10m |
| Infectious Contact (IC) | H | undirected | 410 | 6612 | (445, 352) | 0.042 | 1m |
| Marseilles Contact (MC) | H | undirected | 180 | 4152 | (453, 200) | 0.046 | 1h |
| Student Contact (SC) | H | undirected | 121 | 3608 | (281, 453) | 0.028 | 10m |
| Workplace Contact (WC) | H | undirected | 92 | 1296 | (160, 273) | 0.03 | 1h |
| Reality Mining (RM) | H | undirected | 96 | 17917 | (392, 227) | 0.201 | 1D |
| College Forum (CF) | O | directed | 899 | 12031 | (1134, 135) | 0.079 | 1D |
| College Message (CM) | O | directed | 640 | 15112 | (4413, 155) | 0.022 | 1D |
| Enron Email (EE) | O | directed | 151 | 11204 | (240, 820) | 0.057 | 1D |
| European Research (ER) | O | directed | 254 | 12748 | (271, 316) | 0.149 | 1D |
| Manufacturing Email (ME) | O | directed | 167 | 33673 | (597, 270) | 0.209 | 1D |
| Levant Event (LE) | P | directed | 462 | 54870 | (1011, 307) | 0.177 | 1M |
| Gulf Event (GE) | P | directed | 200 | 49212 | (532, 244) | 0.379 | 1M |
| Aviation Network (AN) | T | directed | 224 | 840881 | (3407, 365) | 0.676 | 1D |
| Britain Transportation (BT) | T | directed | 147 | 282498 | (1282, 360) | 0.612 | 10m |

**Table S1. Basic properties of the real networks.** In the Category column, ‘A’ means animal interactions, ‘H’ represents human contacts, ‘O’ stands for online communications, ‘P’ represents political events, and ‘T’ means transportation networks. In the duration column, ‘D’ represents day, ‘m’ stands for minute, ‘M’ represents month, ‘S’ means second, while ‘h’ refers to hour.

### II. Predictability

Each temporal network can be considered as a random field on the integer lattice$\boldsymbol{Z}^{2}$, i.e. a family of random variables$\left\{ M_{v}:v\in\boldsymbol{Z}^{2} \right\}$, indexed by a two-dimensional vector$v=\left( l,t \right)$. It is natural to assume that each potential link in the temporal network takes weights in a finite set $\mathcal{A}$, i.e.$M_{v}\mathcal{\in A,}v\in\boldsymbol{Z}^{2}$. The uncertainty of a random field can be measured by the entropy rate, which equals the minimum rate required to encode the field without any distortion. Let $M^{LT}$ be the set$\left\{ M_{lt}:1\leq l\leq L,1\leq t\leq T \right\}$, the entropy rate of a random field is defined as

|  | $H\left( M \right)\equiv\lim_{\begin{aligned} L\to\infty\\ T\to\infty\end{aligned}} \frac{1}{LT}H\left( M^{LT} \right)$ |  |
| --- | --- | --- |
|  | $=\lim_{\begin{aligned} L\to\infty\\ T\to\infty\end{aligned}} \frac{1}{LT}\sum_{\begin{aligned} 1\leq l\leq L \\ 1\leq t\leq T \end{aligned}} H\left( M_{lt}\left\vert history of M_{lt} \right. \right)$ |  |
|  | $=\lim_{\begin{aligned} L\to\infty\\ T\to\infty\end{aligned}} \frac{1}{LT}\sum_{\begin{aligned} 1\leq l\leq L \\ 1\leq t\leq T \end{aligned}} H\left( l,t \right)$ | (S1) |

where $M_{lt}$is a certain element in matrix $M$. We use $\Omega_{lt}$to denote the$history of M_{lt}$, i.e.$\Omega_{lt}=\left\{ M_{ij}:\left( j<t \right) or \left( j=t and i<l \right) \right\}$, then $H\left( l,t \right)$is equivalent to$H\left( M_{lt}\left| \Omega_{lt} \right. \right)$.

To calculate the entropy rate, we use the following estimator which has been demonstrated to have good performance over random fields on the integer lattice $\boldsymbol{Z}^{d}$ (*14*)

|  | $\underset{n\to\infty}{liminf} \frac{\sum_{v\in C\left( n \right)} \left( \Lambda_{v}^{v} \right)^{d}}{n^{d}\log n^{d}}\to\frac{1}{H}$ | (S2) |
| --- | --- | --- |

Note that$v=\left( v_{1},v_{2},\ldots,v_{d} \right)$, a $d$-dimensional cube with side k is defined as $M_{C\left( k \right)}$, where$C\left( k \right)=\left\{ v\in\boldsymbol{Z}^{d}:0\leq v_{i}\leq k,for all i \right\}$, $\Lambda_{v}^{v}$ denotes the smallest integer $k$such that block$M_{v-C\left( k \right)}$does not occur within the rectangle$(\boldsymbol{0}, v]$except at position$v$. In our instance, matrix dimension equals two, hence the entropy rate of a temporal network with a large number of snapshots can be estimated as

|  | $H=\frac{n^{2}\log n^{2}}{\sum_{v\in C\left( n \right)} \left( \Lambda_{v}^{v} \right)^{2}}$ | (S3) |
| --- | --- | --- |

Let $P\left( M_{lt}=\hat{M}_{lt}\left| \Omega_{lt} \right. \right)$be the probability that the actual value of$M_{lt}$ agrees with our estimation$\hat{M}_{lt}$, and$\lambda\left( \Omega_{lt} \right)$be the probability that, given$\Omega_{lt}$, $M_{lt}$takes the most likely value, thus

|  | $\lambda\left( \Omega_{lt} \right)\equiv max\left\{ P\left( M_{lt}=\hat{M}_{lt}\left\vert\Omega_{lt} \right. \right) \right\}$ | (S4) |
| --- | --- | --- |

The goal of predictive algorithms is to achieve$\lambda\left( \Omega_{lt} \right)$, i.e. predicting the most likely value $M_{lt}$based on$\Omega_{lt}$.

Next, we define predictability $\Pi_{M}\left( l,t \right)$ for a certain element in random field $M$ based on the history. Let $P\left( \omega_{lt} \right)$be the probability of observing a specific history $\omega_{lt}$, thus

|  | $\Pi_{M}\left( l,t \right)\equiv\sum_{\omega_{lt}} P\left( \omega_{lt} \right)\lambda\left( \omega_{lt} \right)$ | (S5) |
| --- | --- | --- |

The overall predictability$\Pi$of a random field can be obtained by averaging $\Pi_{M}\left( l,t \right)$over all elements, i.e.

|  | $\Pi_{M}\equiv\lim_{\begin{aligned} L\to\infty\\ T\to\infty\end{aligned}}\frac{1}{LT}\sum_{\begin{aligned} 1\leq l\leq L \\ 1\leq t\leq T \end{aligned}} \Pi_{M}\left( l,t \right)$ | (S6) |
| --- | --- | --- |

To calculate the upper bound of predictability$\Pi$, inspired by the method in (*15*), we create a new distribution $P\left( M_{lt}^{'}\left| \omega_{lt} \right. \right)$as random as possible for $P\left( M_{lt}\left| \omega_{lt} \right. \right)$ while preserving the most likely value$\lambda\left( \omega_{lt} \right)=p_{max}$. The rest probabilities are modified to a uniform distribution. Note that$M_{v}\mathcal{\in A}$, and denote $N\equiv\left| \mathcal{A} \right|$. The entropy of the new distribution is

|  | $H\left( M_{lt}^{'}\left\vert\omega_{lt} \right. \right)=-\left( p_{max}logp_{max}+\left( 1-p_{max} \right)log\left( 1-p_{max} \right) \right)+\left( 1-p_{max} \right)log\left( N-1 \right)$ | (S7) |
| --- | --- | --- |

We assume that $\mathcal{F}\left( x \right)=-\left( xlogx+\left( 1-x \right)log\left( 1-x \right) \right)+\left( 1-x \right)log\left( N-1 \right)$, thus $H\left( M_{lt}^{'}\left| \omega_{lt} \right. \right)=\mathcal{F}\left( \lambda\left( \omega_{lt} \right) \right)$ Since $P\left( M_{lt}^{'}\left| \omega_{lt} \right. \right)$ is at least as random as $P\left( M_{lt}\left| \omega_{lt} \right. \right)$, we have

|  | $H\left( M_{lt}\left\vert\omega_{lt} \right. \right)\leq H\left( M_{lt}^{'}\left\vert\omega_{lt} \right. \right)=\mathcal{F}\left( \lambda\left( \omega_{lt} \right) \right)$ | (S8) |
| --- | --- | --- |

Now we use Equation S1, S5 and the concavity of function $\mathcal{F}\left( x \right)$ to obtain the upper bound of predictability $\Pi_{M}$.

$$H\left( M \right)\equiv\lim_{\begin{aligned} L\to\infty\\ T\to\infty\end{aligned}} \frac{1}{LT}\sum_{\begin{aligned} 1\leq l\leq L \\ 1\leq t\leq T \end{aligned}} H\left( M_{lt}\left| \Omega_{lt} \right. \right)=\lim_{\begin{aligned} L\to\infty\\ T\to\infty\end{aligned}} \frac{1}{LT}\sum_{\begin{aligned} 1\leq l\leq L \\ 1\leq t\leq T \end{aligned}} \sum_{\omega_{lt}} P\left( \omega_{lt} \right)H\left( M_{lt}\left| \omega_{lt} \right. \right)$$

$$\leq\lim_{\begin{aligned} L\to\infty\\ T\to\infty\end{aligned}} \frac{1}{LT}\sum_{\begin{aligned} 1\leq l\leq L \\ 1\leq t\leq T \end{aligned}} \sum_{\omega_{lt}} P\left( \omega_{lt} \right)\mathcal{F}\left( \lambda\left( \omega_{lt} \right) \right)\leq\lim_{\begin{aligned} L\to\infty\\ T\to\infty\end{aligned}} \frac{1}{LT}\sum_{\begin{aligned} 1\leq l\leq L \\ 1\leq t\leq T \end{aligned}} \mathcal{F}\left( \sum_{\omega_{lt}} P\left( \omega_{lt} \right)\lambda\left( \omega_{lt} \right) \right)$$

|  | $=\lim_{\begin{aligned} L\to\infty\\ T\to\infty\end{aligned}} \frac{1}{LT}\sum_{\begin{aligned} 1\leq l\leq L \\ 1\leq t\leq T \end{aligned}} \mathcal{F}\left( \Pi_{M}\left( l,t \right) \right)\mathcal{\leq F}\left( \lim_{\begin{aligned} L\to\infty\\ T\to\infty\end{aligned}}\frac{1}{LT}\sum_{\begin{aligned} 1\leq l\leq L \\ 1\leq t\leq T \end{aligned}} \Pi_{M}\left( l,t \right) \right)=\mathcal{F}\left( \Pi_{M} \right)$ | (S9) |
| --- | --- | --- |

Since the function $\mathcal{F}\left( x \right)$ monotonically deceases with $x$, we assume $\mathcal{F}\left( x \right)=H\left( M \right)$, then the value of $x$ in this equation will be no less than $\Pi_{M}$, i.e.,

|  | $H\left( M \right)=-\left( \Pi_{M}^{\max}log\Pi_{M}^{max}+\left( 1-\Pi_{M}^{max} \right)log\left( 1-\Pi_{M}^{max} \right) \right)+\left( 1-\Pi_{M}^{max} \right)log\left( N-1 \right)$ | (S10) |
| --- | --- | --- |

where the $\Pi_{M}^{max}$ in the equation is the upper bound of predictability$\Pi_{M}$, i.e., $\Pi_{M}^{max}\geq\Pi_{M}$.

### III. Generalization and predictive congruency

The entropy rate estimator only applies to square matrix, here we generalize the calculation of predictability for general matrices. Specifically, we split a non-square matrix into a set of squares according to the following rules: Start from left or top of the matrix; Split the largest square from the remaining part; Unit cannot be split (see Fig. S1C). This splitting process is executable and unique for any non-square matrix, and the predictability of this non-square matrix is defined as the weighted average predictability of all the square matrices split from it, where the weight of a square equals the proportion of the square area to the original matrix area.

In order to discover the relationship between the number of squares and the predictability, we continue to successively split all squares into units. To ensure the uniqueness of the splitting procedure, we split the closest square matrix to a set of units to obtain the subsequent phase. We calculate again the predictability for current splitting scheme. The process is repeated until the whole matrix is completely split into units. Suppose the cardinality of finite set $\mathcal{A}$ from which potential links of a temporal network take weights is $\left| \mathcal{A} \right|$, the predictability of a unit is then$\frac{1}{\left| \mathcal{A} \right|}$, since there is not any historical information anymore.

As shown in Fig. S1A, we find a linear relationship between the predictability $p$ and the number of squares *N* (Fig. S1A). Since our measure, the topological-temporal predictability (TTP) corresponds to the value of $p$ when there is only one square split from the original matrix, we can obtain TTP by extending the linear relationship to the dot *N* = 1 (Fig. S1B). We examine the calculation in all other real temporal networks, showing that there are minor deviations in only AN and LE networks (Fig. S6). Suppose matrix $\tilde{M}$ is split into $Q$ squares$s_{1},s_{2},\ldots,s_{Q}$, along with $u$ units in the original splitting phase and the square sizes are$e_{s_{1}},e_{s_{2}},\ldots,e_{s_{Q}}$ respectively, then the predictability acquired at stage $i$ is

$$p_{i}=\left( \sum_{1}^{Q-i+1} {e_{s_{j}}}^{2}p_{s_{j}}+\frac{\sum_{Q-i+2}^{Q} {e_{s_{j}}}^{2}+u}{\left| \mathcal{A} \right|} \right)/D$$

for$i>1$, where $D$ is the total area of $\tilde{M}$. The initial value$p_{1}=\left( \sum_{1}^{Q} {e_{s_{j}}}^{2}p_{s_{j}}+\frac{u}{\left| \mathcal{A} \right|} \right)/D$, and the number of squares is

$$N_{i}=Q-i+1+\sum_{Q-i+2}^{Q} {e_{s_{j}}}^{2}+u$$

since there is a linear relationship between $p_{i}$ and$N_{i}$, we have

$$\frac{p_{i+1}-p_{i}}{N_{i+1}-N_{i}}=k$$

where $k$is a constant and$1\leq i\leq Q-1$. The equation leads to

$$\frac{{e_{s_{i}}}^{2}}{{e_{s_{i}}}^{2}-1}\left( \frac{1}{\left| \mathcal{A} \right|}-p_{i} \right)=kD$$

The left side of this equation remains constant for each square in the original splitting phase. We call this property predictive congruency, which is valid for all real and model networks studied in this paper.

We use predictive congruency to obtain the TTP for any non-square matrix. Indeed, using the linear equation $p_{i}=kN_{i}+b$, we can estimate TTP by extending it to the dot $N_{i}=1$. According to least square regression

$$k=\frac{\left( Q-1 \right)\sum_{1}^{Q-1} \left( N_{i}p_{i} \right)-\sum_{1}^{Q-1} N_{i}\sum_{1}^{Q-1} p_{i}}{\left( Q-1 \right)\sum_{1}^{Q-1} {N_{i}}^{2}-\left( \sum_{1}^{Q-1} N_{i} \right)^{2}}$$

$$b=\frac{\sum_{1}^{Q-1} p_{i}-k\sum_{1}^{Q-1} N_{i}}{Q-1}$$

hence, TTP for any non-square matrix is

$$p{=p}_{N_{i}=1}=k+b=\frac{\left( Q-1 \right)\sum_{1}^{Q-1} \left( N_{i}p_{i} \right)-\sum_{1}^{Q-1} N_{i}\sum_{1}^{Q-1} p_{i}}{\left( Q-1 \right)\sum_{1}^{Q-1} {N_{i}}^{2}-\left( \sum_{1}^{Q-1} N_{i} \right)^{2}}+\frac{\sum_{1}^{Q-1} p_{i}-k\sum_{1}^{Q-1} N_{i}}{Q-1}$$




Figure. S1. The splitting process for a non-square matrix and the calculation of predictability. The dataset HP is used as an example. **(A)** N is the number of squares (including units), $p$ is the weighted average predictability of the whole matrix in the splitting phase. Each point corresponds to a splitting phase and the line represents regression. **(B)** The area that approaches axis is zoomed in, where we use the linear relationship to obtain TTP of a matrix. **(C)** Different phases generated by the splitting process. Each matrix corresponds to a splitting phase, the color of each square represents the splitting order: Green denotes the first square to split in next phase, orange is the last square to split while blue represents units. In this instance, we obtain six predictabilities from corresponding splitting phases.





Figure. S2. The relationship between predictability $p$ and the number of squares *N* for 18 real temporal network datasets. Each point corresponds to a splitting phase and the lines represent respective regression.

### IV. Matrix shuffling and filtering

To obtain $\mathrm{TTP}_{\mathrm{baseline}}$, we usually need to use the average of many different realizations of shuffled network to diminish the errors, yet considering the high time complexity of $\mathrm{TTP}$, we attempt to find a minimum number of realizations that can roughly achieve the same goal. We obtain 100 different shuffling of a network, and average over 100 different combinations of those realizations. Figure S3 indicates the stabilization after we average over 40 different realizations of shuffled network, thus we adopt the average of at least 40 runs as the $\mathrm{TTP}_{\mathrm{baseline}}$ of the network.





Figure. S3. Average of $\mathrm{TTP}_{\mathrm{baseline}}$ against the number of realizations. A synthetic unweighted network with rewiring probability as 0.1 is used as an example.

Due to the extremely high sparsity of real world networks, we use matrix filtering to remove node pairs that never or seldom have connections (See Methods). By changing the portion of matrix being used, we find that although $\mathrm{TTP}$ and $\mathrm{TTP}_{\mathrm{baseline}}$ increase due to more inactive links being included, matrix filtering has no impact on $N\mathrm{TTP}$ after 30% of most active links are included in the matrix. We continue to remove inactive links from the remaining matrix of real world networks and find that $N\mathrm{TTP}$ maintains stable at an early stage (Sec. VII.).





Figure. S4. Impact of portion of active links on $\mathrm{TTP}$, $\mathrm{TTP}_{\mathrm{baseline}}$and $N\mathrm{TTP}$. A synthetic unweighted network with rewiring probability as 0.1 is used as an example.

### V. Impact of row orders

Different orders of rows in matrix $\tilde{M}$ might result in different values of entropy rate. In this section, we systematically examine the impact of row orders.

First, we use Genetic Algorithm (GA) to obtain the specific order of rows that achieve the highest or lowest predictability. In GA, the row order is encoded as the chromosome, and the $\mathrm{TTP}$ serves as the fitness function (*16*). Assume that there are $n$ rows in the matrix and the ordering sequence is $S=\left( s_{1}, s_{2},\ldots,s_{n} \right)$, where $s_{i}$ represents the sequence number of row $i$ and apparently $s_{i}\in\left\{ 1,2,\ldots,n \right\};s_{i}\neq s_{j} for i\neq j, i,j\in\left\{ 1,2,\ldots,n \right\}$. The genetic sequence is denoted by $G=\left( g_{1}, g_{2},\ldots,g_{n} \right)$, and $Y=\left( y_{1}, y_{2},\ldots,y_{n} \right)$ represents the ascending sequence ($1, 2,\ldots, n)$, then the encoding process is as follows:

1. Suppose $y_{j}=s_{i}$, and the sequence number of $y_{j}$ in $Y$ is $x$, then $g_{i}=x$;
2. Delete $y_{j}$ from $Y$;
3. Loop through 1) and 2) until the end.

Since $g_{i}\in\left\{ 1, 2,\ldots, n-i+1 \right\}$, in the crossover operations we exchange only the corresponding part of two chromosomes, and in mutation operations we mutate only the gene in its value range. Let $D=\left( d_{1}, d_{2},\ldots,d_{n} \right)$ be the decoding sequence, the decoding process is quite similar to encoding:

1. Suppose $y_{j}$ is the $g_{i}$-th element in $Y$, then $d_{i}=y_{j}$;
2. Delete $y_{j}$ from $Y$;
3. Loop through 1) and 2) until the end.

The parameters are population size = 50, generation = 100, crossover probability = 0.5, mutation probability = 0.001. In order to retain good individuals with a higher probability in the selection process, we use exponential function to enlarge the difference between good and bad individuals. The goal of genetic algorithm is to obtain not only$\max\left( p \right)$, the maximum value of $\mathrm{TTP}$, but also$\min\left( p \right)$, the minimum value of $\mathrm{TTP}$. We extract a group of submatrices with different sizes from a real temporal network AN and observe the variations in $\max\left( p \right)$ and$\min\left( p \right)$, as well as $\mathrm{TTP}$ of 100 random orders.


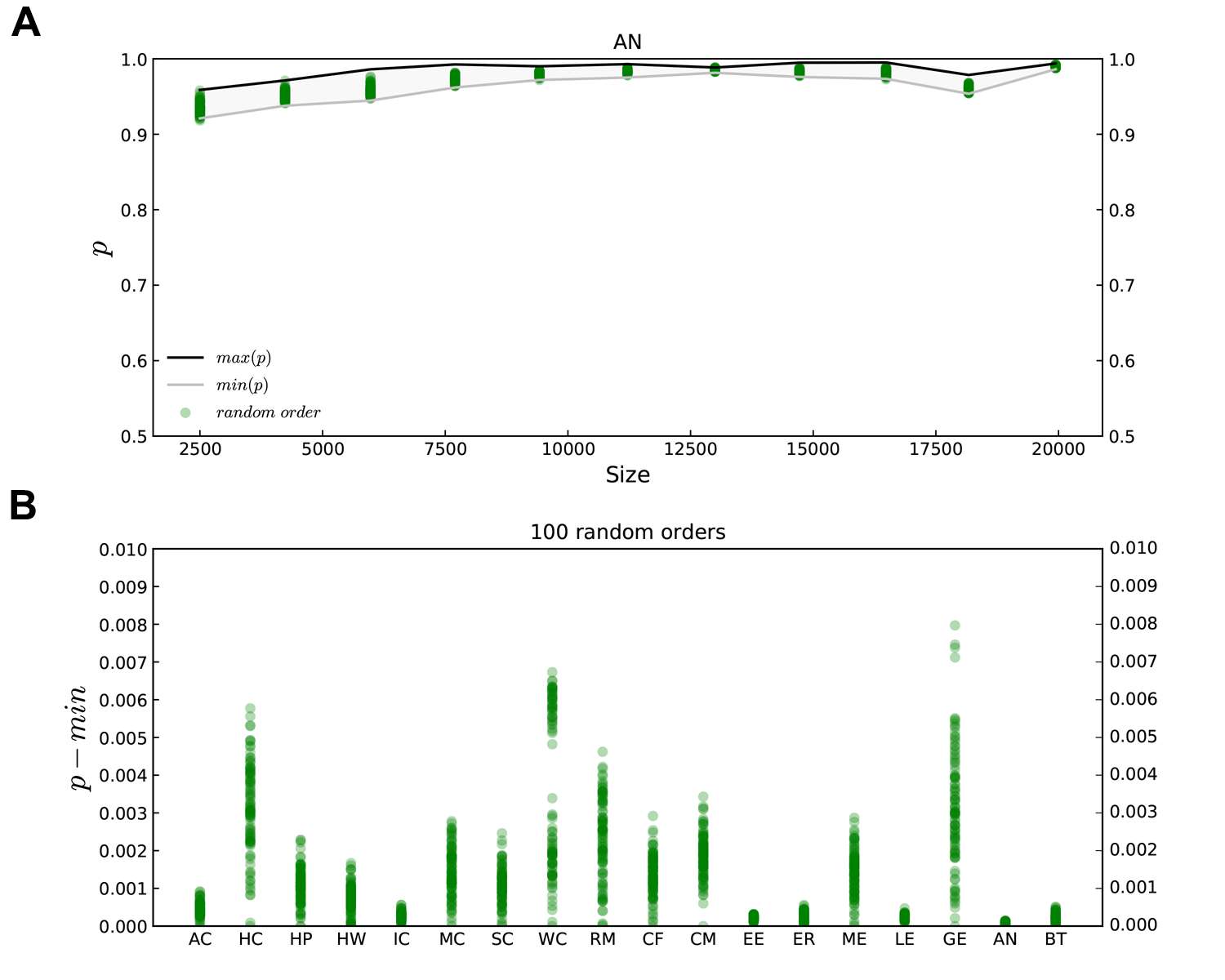


Figure. S5. **(A)** $max\left( p \right)$ and$min\left( p \right)$ against the size of submatrices extracted from dataset AN. The results of 100 submatrices with random row orders are also provided for comparison. **(B)** We reorder the rows in each dataset of 18 real temporal networks and display the corresponding predictabilities. The results show that the difference in predictability is small (<0.004 for most networks, and <0.008 for all networks).

Figure S5A exhibits the lowest and highest $\mathrm{TTP}$s obtained by genetic algorithm, as well as $\mathrm{TTPs}$ for random row orders. We find that the gap between the lowest and the highest $\mathrm{TTPs}$ are small and 100 random row orders are enough to sample the $\mathrm{TTPs}$ within the gaps. Hence, we sample 100 random row orders for all 18 real temporal networks and test the fluctuations in $\mathrm{TTPs}$. As shown in Fig. S5B, the $\mathrm{TTPs}$ of a certain network changes little for different orders of rows in the corresponding matrix. Indeed, for all 18 networks the largest fluctuations are less than 0.008, and for 15 of the networks the largest fluctuations are even less than 0.004. Since $\mathrm{TTPs}$ represents the upper bound of predictability of a temporal network, in the paper we calculate $\mathrm{TTPs}$ as the maximal predictability in 100 random sorted realizations of the matrix $\tilde{M}$.

To further test the fluctuation in $\mathrm{TTP}$ against row order changes on the first synthetic model, we adjust rewiring probability and observe the similar pattern of variation. Increase in both dimension of size leads to the decline in $\mathrm{TTP}$ fluctuation, meanwhile the fluctuation remains consistently lower than 0.008, indicating that $\mathrm{TTP}$ is independent of row orders of the matrix.





Figure. S6. Fluctuation in $\mathrm{TTP}$ on the first synthetic model, with rewiring probability from 0 to 1.0.

### VI. Impact of snapshot duration

To test the impact of time window on $\mathrm{TTP}$ and $N\mathrm{TTP}$, we obtain a group of temporal networks by applying different snapshot duration to real datasets.





Figure. S7. (Top) TTP and link density vs the number of snapshots. (Bottom) NTTP and baseline vs the number of snapshots.

For datasets ME and RM, there are fluctuations in $\mathrm{TTP}$ and $N\mathrm{TTP}$ when the snapshot duration changes, but their varying patterns are quite similar (Fig. S7). When snapshot duration becomes smaller and the number of snapshots increases, there is a short decline then a steady increase in $\mathrm{TTP}$. The varying patterns of $\mathrm{TTP}$ and $N\mathrm{TTP}$ are more similar on ME, while $N\mathrm{TTP}$ of RM fluctuates within a small interval. $\mathrm{TTP}$ changes in the opposite trend with link density, mainly due to the more predictable nature of more sparse network, especially for the same network with different time windows. Baselines are quite close to $\mathrm{TTP}$ in both datasets, implying that network sparsity is the most significant contributor to the high predictability. In this paper, we adopt the most frequently used snapshot duration for each real network.

### VII. NTTP of incomplete data

To test the impact of data incompleteness on the predictability of temporal networks, we remove a proportion of links in each real dataset and calculate its NTTP.


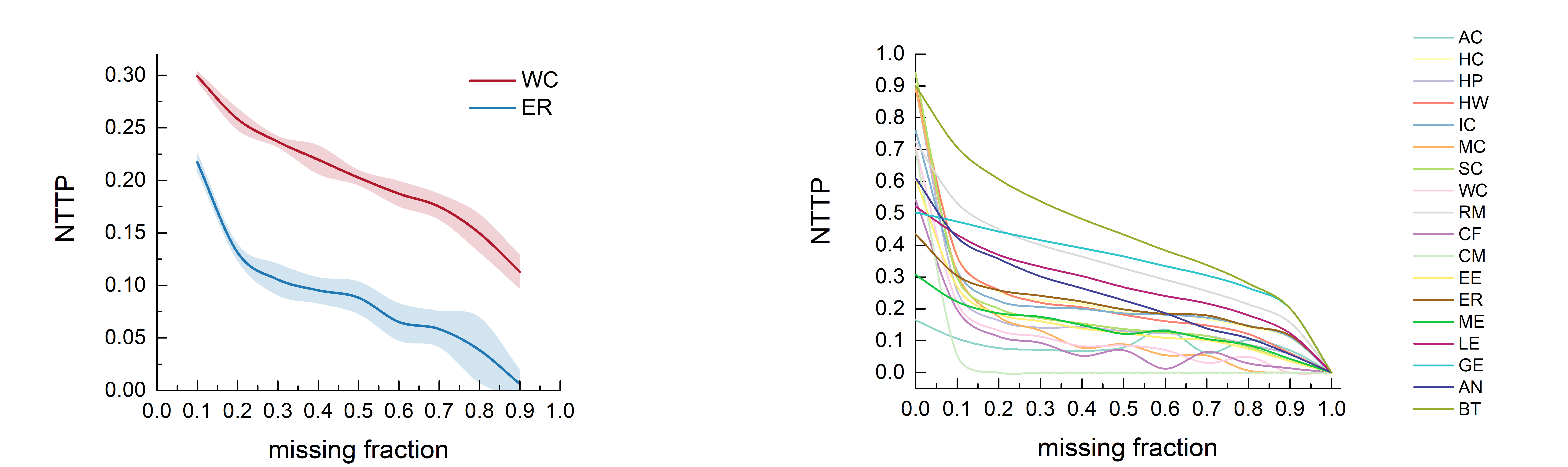


Figure. S8. (Left) NTTP against the fraction of missing links for datasets WC and ER. (Right) NTTP vs missing fraction for all datasets.

As shown in Fig. S8, for most real networks NTTP decreases rapidly with the proportion of links that are removed from datasets. Especially for large networks, such as CM, the predictability decreases to nearly zero when 10% links are removed. The fundamental reason for this phenomenon is that most real temporal networks are extremely sparse. Indeed, each snapshot is usually a sparse network and the expanded matrix that describes all snapshots has only a very small fraction of non-zero entries. The predictability of a temporal network is based only on the patterns of such non-zero entries (existing links). If some of such a small fraction of non-zero entries are removed, the information encoded in the link pattern diminishes quickly. When the network is not too sparse, such as EE, its predictability decreases relatively slowly with the proportion of missing links.

### VIII. NTTP of Submatrices





Figure. S9. **(A)** NTTP of a submatrix vs its temporal proportion in the matrix. **(B)** NTTP of a submatrix vs its topological proportion in the matrix. **(C)** NTTP of the whole network and NTTP of sliding window submatrix containing 40% of consecutive observation period. **(D)** NTTP of the whole network and NTTP of sliding window submatrix containing 40% of adjacent links.

In this section we examine the predictability of a part of a temporal network. Figures S9A, B indicate that we can obtain $N\mathrm{TTP}$ of the whole network even considering only 70% of adjacent elements in a temporal network or only 50% of the snapshots. Figures S8C, D exhibit the average $N\mathrm{TTP}$ while considering temporal submatrices and topological submatrices that contain only 40% snapshots and adjacent links respectively. We find that the $N\mathrm{TTPs}$ of 9 (6) real networks are within the predictability intervals of their temporal (topological) submatrices. We further show in Figs. S10B, D that, for networks with large temporal length and link density $N\mathrm{TTP}$ can be well estimated through topological submatrices, while for networks with large topological length and link density $N\mathrm{TTP}$ can be well estimated through temporal submatrices.

These results indicate that the predictability of a real temporal network can be well estimated from its submatrices. Especially, when the network is extremely large, using submatrices to calculate $N\mathrm{TTP}$ can dramatically reduce time complexity at the cost of less than 5% error for all the datasets studied in this paper.





Figure. S10. **(A)** Error of NTTP estimation from temporal submatrix. **(B)** Distribution of datasets in 2-D space indexed by density and topological length. **(C)** Error of NTTP estimation from topological submatrix. **(D)** Distribution of datasets in 2-D space indexed by density and temporal length. Red lines or dots are datasets with large error, black lines or dots refer to networks with relative minor error, and blue lines are mean results of black lines.

### IX. Characteristics of real temporal networks

To determine the cause of high predictability we study the correlation between $\mathrm{TTP}$ and $\mathrm{TTP}_{\mathrm{baseline}}$. Interestingly, $\mathrm{TTP}$ is proportional to the $\mathrm{TTP}_{\mathrm{baseline}}$ for both model and real networks, indicating that theoretically there is significant room to improve the quantification of predictability. Furthermore, the high predictability mostly comes from the distribution of link weights, i.e. the sparsity of networks, additionally revealing the significance of adopting normalized predictability for analyzing intrinsic predictable nature of temporal networks.





Figure. S11. Correlation between $\mathrm{TTP}$ and the $\mathrm{TTP}_{\mathrm{baseline}}$ for real networks as well as temporal stochastic block model.

While there seems to be no significant pattern in animal interactions and political events, activities of human contacts are quite bursty and highly synchronized (Figure S12). It’s not difficult to understand the fundamental cause of it lies in the regular nature of human life. Less restricted by space and distance than proximity networks, online communications are considerably less bursty and synchronized, leading to generally lower predictability. Periodicity of BT contributes to its highly predictable nature, while less regular pattern of AN defines its more random essence.





Figure. S12. Number of events in single snapshot with time. Since lengths of time window for networks are different, we use normalized time index for each network. Color of lines corresponds to value of NTTP, while color of legends in each subplot represents its type.

Even though periodic pattern is only observed on human-related networks (human contacts, online communications and transportation) in Figure S12, we discover all the real networks are highly synchronized (See Figure S13), implicating the contacts or events tend to happen at the same time. The symmetry and local maximum also accord perfectly with the observations in Figure S11, strengthening again the periodicity of real networks.


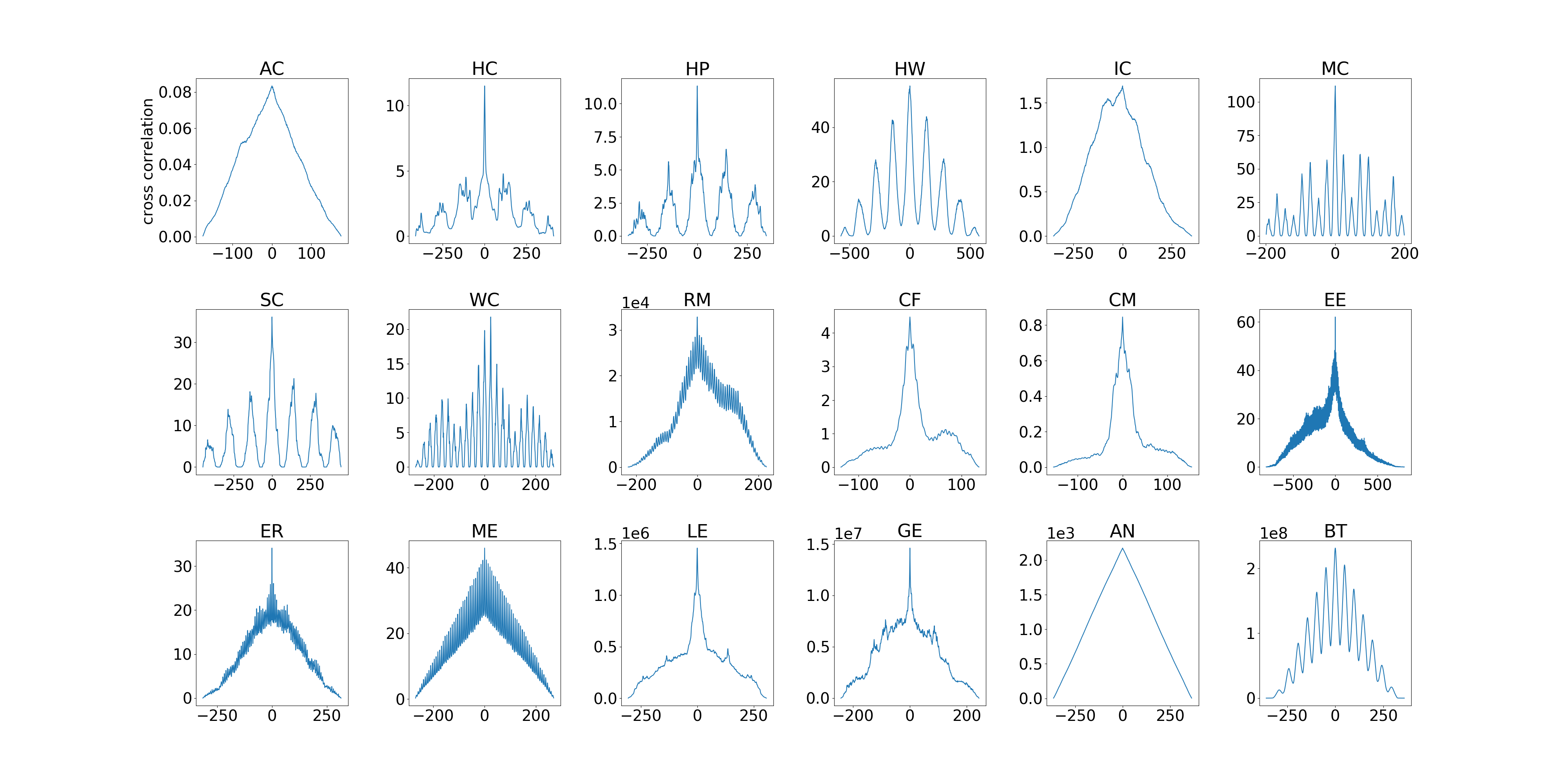


Figure. S13. Cross correlations of all link pairs on real networks. Since the time length of each network can be different, the range of displacement also differs among networks.

Although there’s no significant correlation between NTTP and burstiness for real networks (See Figure S14), human contacts have the highest predictability with the most bursty nature among all networks. Based on the characteristics of real networks, burstiness is one of the main causes of the highly predictable nature of human contacts.


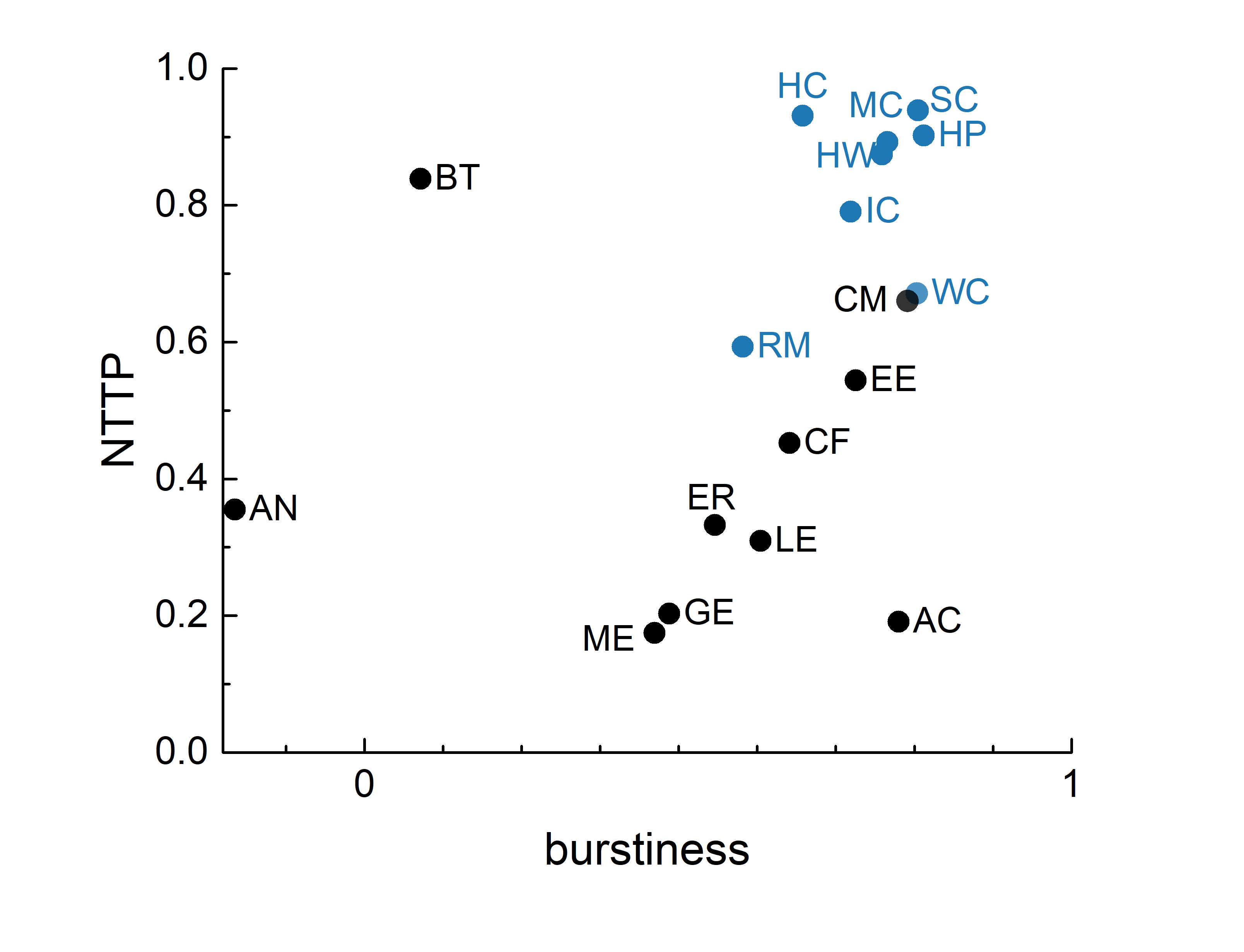


Figure. S14. NTTP vs burstiness on real world networks. Human contact networks are marked as blue dots.

### X. Graphic presentation of model networks

To observe the evolving patterns of model networks introduced in Fig. 2 B and C, we show the visualization of them in Figure S15 and 16. For the neighbor correlation model, the larger the $\beta$ and $\gamma$, the higher the TTP. Since $\beta$ and $\gamma$ are not independently controlling the memory strength, the network becomes much more predictable when they are simultaneously increasing. Despite the fact that $\beta$ and $\gamma$ controls topological and temporal correlation respectively, generated network has displayed diagonal memory. The varying pattern of long-range correlation model is quite similar. The variation trend of TTP corresponds with the regularity of network, demonstrating TTP’s validity.


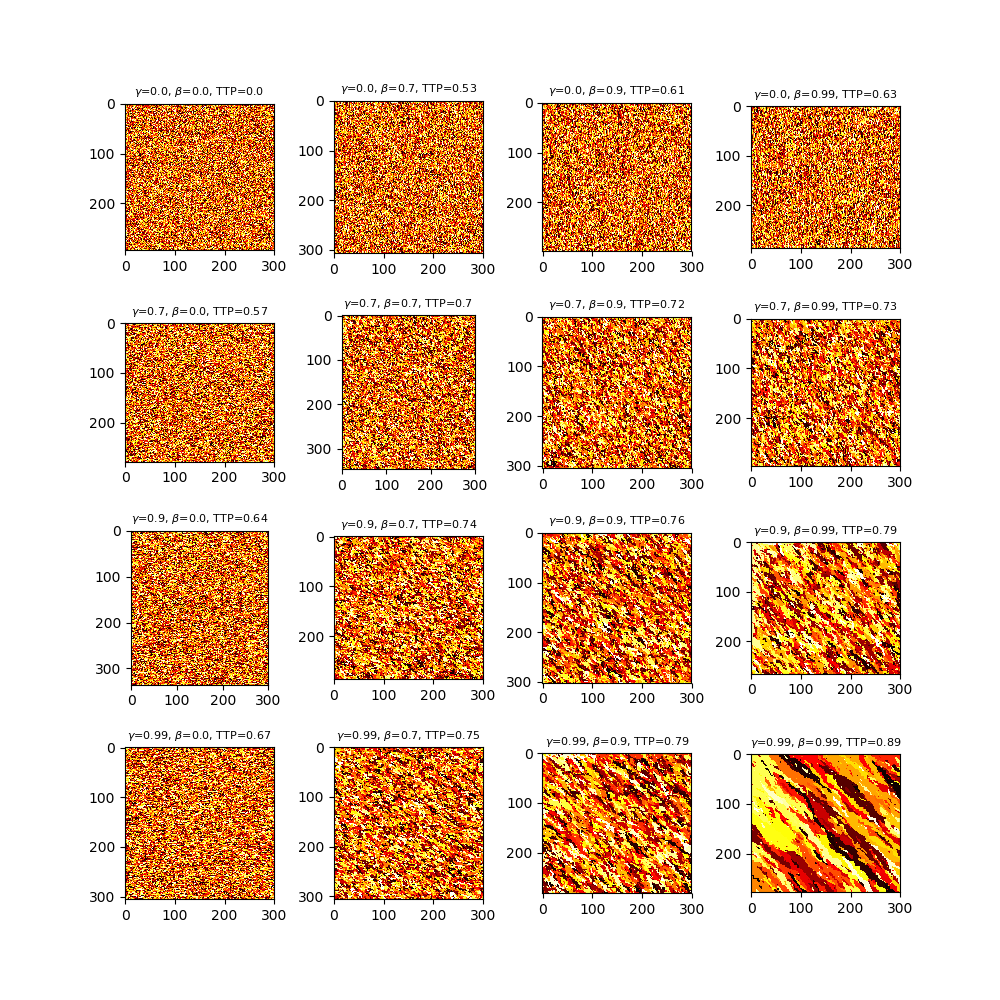


Figure. S15. Visualization of neighbor correlation model. Topological parameter $\beta$ and temporal parameter $\gamma$ determines the memory strength in each dimension.


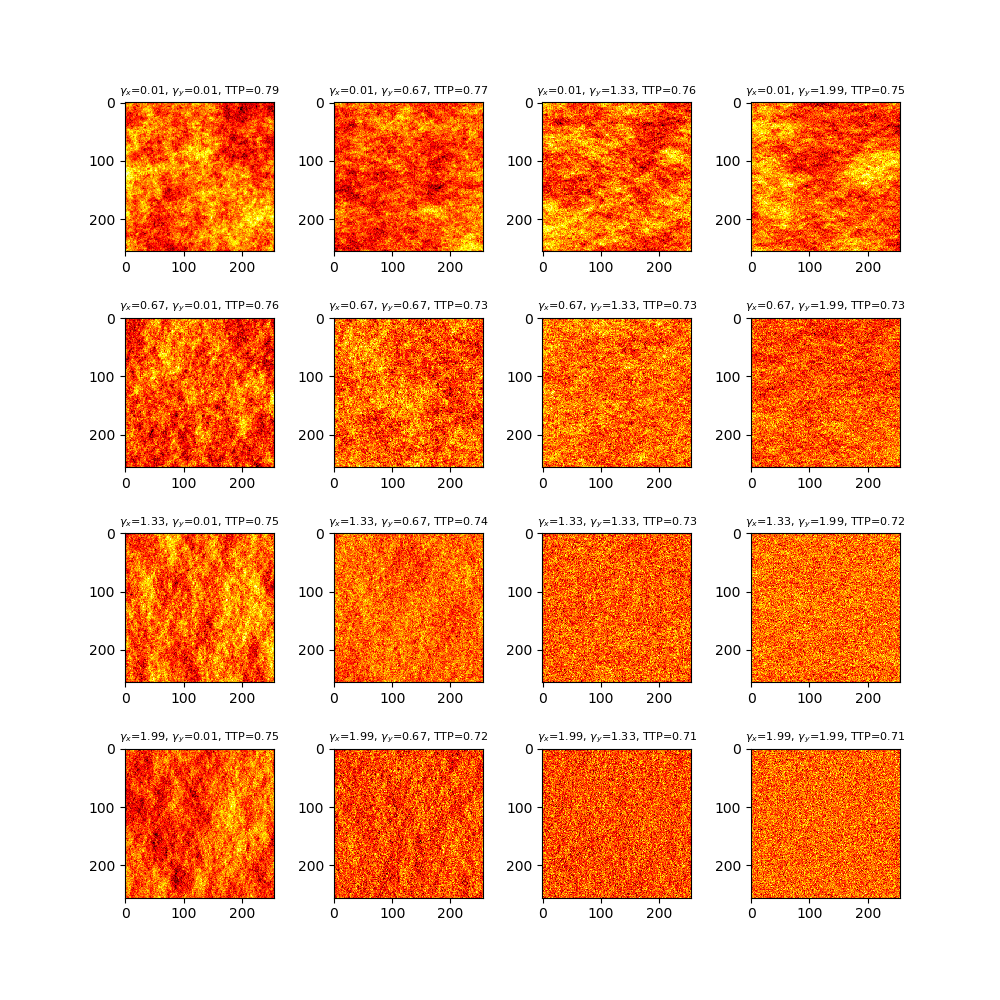


Figure. S16. Visualization of long-range correlation model. $\gamma_{x}$ and $\gamma_{y}$ are regarded as decay rates in temporal and topological dimension respectively.

### XI. Predictive algorithms

Markov is a predictive algorithm that considers a temporal network as a set of uncorrelated time series, and only uses the temporal information of each time sequence to predict. Suppose the time series is $S$, its length is $T$, and set the memory length as $l$, then the time series is divided into $T-l$ groups of time series, of which the i-th group consists of $\left\{ S_{i}, S_{i+1}, \ldots,S_{i+l} \right\}$, with $\left\{ S_{i},S_{i+1},{\ldots,S}_{i+l-1} \right\}$ being the history to help us predict $S_{i+l}$. These $l$-length groups are randomly shuffled since there are no intergroup correlations under this circumstance, and 70% of the groups are used as training set while 30% are test set.

For each link in the temporal network we construct an $l$-th order transition matrix, in which the rows are the input states of $\left\{ S_{i},S_{i+1},{\ldots,S}_{i+l-1} \right\}$, and columns are the output states of the next timestamp $S_{i+l}$. We modify the transition matrix through training set, and use the test set to check its performance. When predicting a time series, previous $l$ symbols prior to the object determine the input state, and the output state with the highest transition probability in the matrix is the output of the algorithm.

The Long Short-Term Memory network (LSTM) (*17*) is considered as an effective model to process sequential data, which is used as a basic model in this paper. The most significant difference between predicting time series and predicting network is that there is some correlation among links in network which could be used to promote prediction. So we adopt convolution LSTM (ConvLSTM) (*18*), an improved version of the LSTM network, which captures spatiotemporal correlations of a network rather than just its temporality.

Assume there are $T$ snapshots in a temporal network, and the history length is set as$s$, which means we use preceding$s$snapshots to predict the next one. As a result we obtain$T-s$ groups of network packages for prediction. For each network package, former$s$snapshots are used as the input of ConvLSTM, with the last one as the output, or the label of ConvLSTM, since it is supervised learning. 70% of the network packages is used as the training set and 30% as the test set.

For ConvLSTM, the input $X_{1}, X_{2},\ldots, X_{t}$, cell outputs $C_{1}, C_{2},\ldots, C_{t}$, hidden states $H_{1}, H_{2},\ldots, H_{t}$, and gates $i_{t}, f_{t}, o_{t}$ are three-dimensional tensors, while the counterparts of the FC-LSTM are two-dimensional vectors. Therefore we transform the two-dimensional $1\times N^{2}$ matrix into a three-dimensional $1\times N\times N$ matrix as the input. The ConvLSTM determines the future state of a certain cell in the grid by the inputs and previous states of its local neighbors, which can be easily achieved by using a convolution operator in the state-to-state and input-to-state transitions (see Figure S17).

The ConvLSTM cell is the same as LSTM cell (see Figure S17). The key equations of ConvLSTM are shown below, where ‘*’ denotes the convolution operator and ‘$\circ$’ denotes the Hadamard product:

$$i_{t}=\sigma\left( W_{xi}*X_{t}+W_{hi}*H_{t-1}+W_{ci}\circ C_{t-1}+b_{i} \right)$$

$$f_{t}=\sigma\left( W_{xf}*X_{t}+W_{hf}*H_{t-1}+W_{cf}\circ C_{t-1}+b_{f} \right)$$

$$C_{t}=f_{t}\circ C_{t-1}+i_{t}\circ tanh\left( W_{xc}*X_{t}+W_{hc}*H_{t-1}+b_{c} \right)$$

$$o_{t}=\sigma\left( W_{xo}*X_{t}+W_{ho}*H_{t-1}+W_{co}\circ C_{t-1}+b_{o} \right)$$

$$H_{t}=o_{t}\circ tanh\left( C_{t} \right)$$

While the key equations of FC-LSTM are shown in below:

$$i_{t}=\sigma\left( W_{xi}x_{t}+W_{hi}h_{t-1}+W_{ci}\circ c_{t-1}+b_{i} \right)$$

$$f_{t}=\sigma\left( W_{xf}x_{t}+W_{hf}h_{t-1}+W_{cf}\circ c_{t-1}+b_{f} \right)$$

$$C_{t}=f_{t}\circ c_{t-1}+i_{t}\circ tanh\left( W_{xc}x_{t}+W_{hc}h_{t-1}+b_{c} \right)$$

$$o_{t}=\sigma\left( W_{xo}x_{t}+W_{ho}h_{t-1}+W_{co}\circ c_{t-1}+b_{o} \right)$$

$$h_{t}=o_{t}\circ tanh\left( C_{t} \right)$$

We use one hidden layer in ConvLSTM model, and feature preprocess is necessary before training. Feature vectors are normalized using scaler function and transformed through reshape function. We select Relu function as our activation function and Adam algorithm as the optimization function.

Since each link of the network takes value in a discreet set, the output of our predicting algorithm should also be discretized. Therefore before predicting, we discretize our prediction as its closest element in the value set $W$ of the network. Suppose $P$ equals the number of correct predictions, while $N$ is the number of incorrect ones, then the accuracy of prediction for series $m$ is defined as$a_{m}=\frac{P}{P+N}$, thus the accuracy of predicting the whole network is$a=\frac{\sum_{\boldsymbol{m}}^{M} a_{m}}{M}$.

The Predictive Coding Network (PredNet) (*19*) is a deep convolutional recurrent neural network inspired by the principles of predictive coding from the neuroscience literature. It is trained for next-frame video prediction with the belief that prediction is an effective objective for unsupervised learning. We adopt a three-layer PredNet model as a predictive algorithm in this paper, and the input and data preprocessing are the same as the counterparts of ConvLSTM.


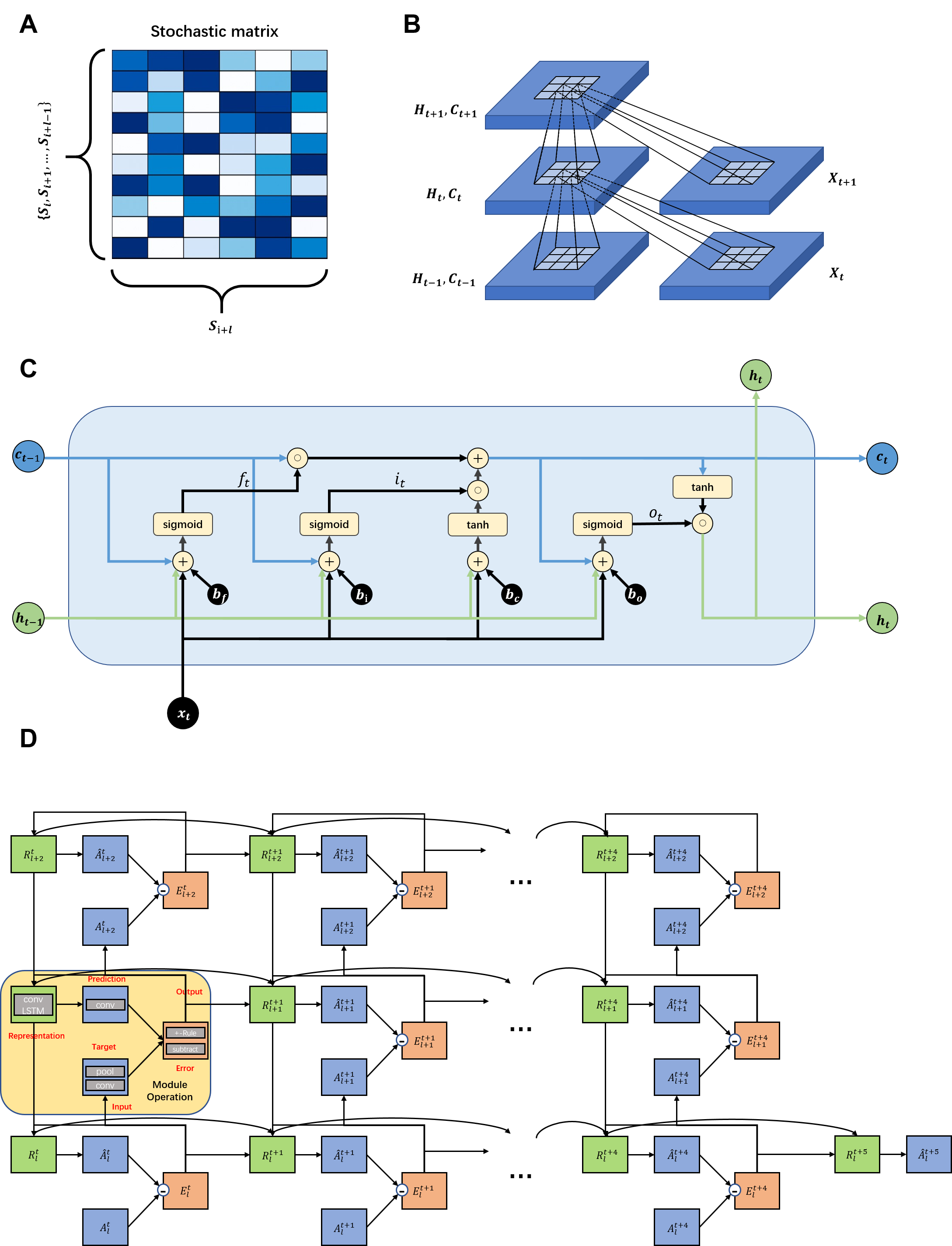


Figure. S17. (a) Transition matrix after training. Each row in the matrix corresponds to an input state vector $\left\{ S_{i}, S_{i+1},\ldots,S_{i+l-1} \right\}$, and each column is the transition probability to an output state $S_{i+l}$. (b) Inner structure of ConvLSTM, $X$ is the input while $H$ and $C$ are the parameters. (c) Structure of ConvLSTM cell. (d) Flow diagram of PredNet. $S_{i}, S_{i+1},\ldots,S_{i+l-1}$ are the input states and $S_{i+l}$ is the predicted state.

### References

1. Blonder, B., & Dornhaus, A. Time-Ordered Networks Reveal Limitations to Information Flow in Ant Colonies. *PloS One* 2011; **6**: e20298.
2. Gallotti, R., & Barthelemy, M. The multilayer temporal network of public transport in Great Britain. *Scientific Data* 2015; **2**: 140056.
3. Opsahl, T. Triadic closure in two-mode networks: Redefining the global and local clustering coefficients. *Social Networks* 2013; **35**: 159-167.
4. Panzarasa, P., Opsahl, T., & Carley, K. M. Patterns and dynamics of users' behavior and interaction: Network analysis of an online community. *Journal of the American Society for Information Science and Technology* 2009; **60**: 911-932.
5. Shetty, J., & Adibi, J. The Enron email dataset database schema and brief statistical report. *Information sciences institute technical report, University of Southern California* 2004; **4**: 120-128.
6. Paranjape, A., Benson, A. R., & Leskovec, J. Motifs in temporal networks. *In Proceedings of the Tenth ACM International Conference on Web Search and Data Mining* (601-610). ACM, 2017.
7. Michalski, R., Palus, S., & Kazienko, P. Matching organizational structure and social network extracted from email communication. *In International Conference on Business Information Systems* (197-206). Berlin : Springer, 2011.
8. Chaintreau, A. *et al*. Impact of human mobility on opportunistic forwarding algorithms. *IEEE Transactions on Mobile Computing* 2007; **6**: 606-620.
9. Isella, L. *et al*. What's in a crowd? Analysis of face-to-face behavioral networks. *Journal of Theoretical Biology* 2011; **271**: 166-180.
10. Vanhems, P. *et al*. Estimating potential infection transmission routes in hospital wards using wearable proximity sensors. *PloS One* 2013; **8**: e73970.
11. Fournet, J., & Barrat, A. Contact patterns among high school students. *PloS One* 2014; **9**: e107878.
12. Eagle, N., & Pentland, A. Reality mining: sensing complex social systems. *Personal and Ubiquitous Computing* 2006; **10**: 255-268.
13. Génois, M. *et al*. Data on face-to-face contacts in an office building suggest a low-cost vaccination strategy based on community linkers. *Network Science* 2015; **3**: 326-347.
14. Kontoyiannis, I., Algoet, P. H., Suhov, Y. M., & Wyner, A. J. Nonparametric entropy estimation for stationary processes and random fields, with applications to English text. *IEEE Transactions on Information Theory* 1998; **44**: 1319-1327.
15. Song, C., Qu, Z., Blumm, N., & Barabási, A.L. Limits of predictability in human mobility. *Science* 2010; **327**: 1018-1021.
16. Deb, K., Pratap, A., Agarwal, S., & Meyarivan, T. A. M. T. A fast and elitist multiobjective genetic algorithm: NSGA-II. *IEEE Transactions on Evolutionary Computation* 2002; **6**: 182-197.
17. Hochreiter, S., & Schmidhuber, J. Long short-term memory. *Neural computation* 1997; **9**: 1735-1780.
18. Xingjian, S. *et al*. Convolutional LSTM network: A machine learning approach for precipitation nowcasting. *In Advances In Neural Information Processing Systems* 802-810, 2015.
19. Lotter, W., Kreiman, G., & Cox, D. Deep predictive coding networks for video prediction and unsupervised learning. arXiv preprint arXiv:1605.08104, 2016.
